# UCHL3 Regulates Subgenomic Flaviviral RNA Condensates to Promote Virus Propagation

**DOI:** 10.1002/advs.202521781

**Published:** 2026-06-03

**Authors:** Oscar Trejo‐Cerro, Anna Beekmayer‐Dhillon, Qi Wen Teo, Lewis Siu, Ming Yuan Li, Kate J. Heesom, Adán Pinto‐Fernández, Sumana Sanyal

**Affiliations:** ^1^ Sir William Dunn School of Pathology University of Oxford Oxford UK; ^2^ HKU‐Pasteur research pole School of Public Health University of Hong Kong Hong Kong SAR China; ^3^ Department of Chemical Pathology and Li Ka Shing Institute of Health Sciences Chinese University of Hong Kong Hong Kong SAR China; ^4^ Proteomics Facility University of Bristol Bristol UK; ^5^ Translational Ubiquitomics Laboratory Nuffield Department of Medicine CAMS Oxford Institute and Centre for Medicines Discovery University of Oxford Oxford UK; ^6^ Current address: Department of Biochemistry University of Illinois Urbana‐Champaign Urbana IL USA

**Keywords:** biomolecular condensates, dengue, deubiquitylases, sfRNA, Zika

## Abstract

Flavivirus subgenomic RNAs (sfRNAs) antagonise antiviral defences, yet how sfRNAs are organized and maintained in cells remains poorly understood. Here we identify ubiquitin C‐terminal hydrolase L3 (UCHL3) as a post‐translational regulator of flavivirus sfRNA stability and function. Using activity‐based protein profiling during ZIKV and DENV infections, we discovered that UCHL3 is activated upon flavivirus infection. CRISPR‐Cas9 knockout of UCHL3 significantly impaired viral replication and reduced viral protein expression across multiple cell models; reconstitution with wild‐type UCHL3 rescued these defects, whereas a catalytically inactive mutant (C95A) failed to restore replication, confirming the requirement for deubiquitylase activity. Through a proximity‐biotinylation sfRNA‐interactome capture assay, we show that UCHL3 physically interacts with sfRNA complexes. Importantly, UCHL3 deletion accelerates RNase L activation, causing enhanced sfRNA relocalization from protective P‐bodies to PABPC1‐positive RNase L‐induced bodies (RLBs), resulting in viral RNA decay. RNase L knockdown restores viral replication in UCHL3‐deficient cells, confirming RNase L‐dependent antiviral effects. This pro‐viral effect of UCHL3 operates through interferon‐independent mechanisms, as replication defects persist despite exogenous interferon treatment. This work therefore identifies UCHL3 as a regulator of sfRNA fate between pro‐viral and antiviral RNA condensates, identifying a post‐translational mechanism governing viral RNA stability and a potential therapeutic target for flavivirus infections.

## Abstract 2

Flaviviruses exploit host machinery to evade innate immunity, yet the post‐translational mechanisms governing viral RNA stability remain unclear. This study identifies the deubiquitylase UCHL3 as a pro‐viral factor that suppresses RNase L activation and maintains subgenomic flaviviral RNA within protective P‐body condensates. Loss of UCHL3 redirects viral RNA to degradative compartments, impairing replication. UCHL3 represents a potential therapeutic target for flavivirus infections.

## 1 Introduction

Flaviviruses, such as Zika virus (ZIKV), dengue virus (DENV), and West Nile virus (WNV), pose significant global health threats on account of their ability to cause severe neurological complications, haemorrhagic fever, and birth defects [1]. These positive‐sense RNA viruses have evolved intricate strategies to hijack cellular machinery for efficient replication while evading innate immune responses [2].

A hallmark of flavivirus transmissibility and their epidemic potential is the generation of subgenomic flavivirus RNA (sfRNA), a highly structured noncoding RNA produced when the host 5'‐3' exoribonuclease XRN1 stalls on RNA secondary structures during viral genome degradation [3, 4]. This conserved feature across the flavivirus family serves as a virulence determinant. Multiple lines of evidence show that viruses unable to produce sfRNAs replicate less efficiently, display reduced pathogenesis in animal models, and cause diminished cytotoxicity in cell culture [5, 6]. Consequently, sfRNAs have evolved as an essential component of flavivirus replication strategies and immune evasion.

Emerging evidence indicates several potential functions of sfRNA in both the human and vector hosts. These include antagonism of antiviral immunity, promotion of replication, viral fitness and host adaptation [5, 6]. These molecules are thought to function as molecular decoys that sequester RNA‐binding proteins and interfere with antiviral signaling pathways [7]. For example, sfRNAs can mimic natural substrates for Dicer and Argonaute, thereby interfering with antiviral RNAi responses in insect cells [8]. Recent evidence has revealed that sfRNAs organize into dynamic ribonucleoprotein complexes resembling stress granules, forming biomolecular condensates that concentrate viral and cellular factors essential for replication and immune evasion [9]. These sfRNA‐containing condensates appear to be distinct from classical stress granules: they are smaller in size (∼1µm), and colocalize with G3BP1, PABPC1, UBAP2L, and poly(A) RNA [9].

The formation, composition, and dynamics of biomolecular condensates can be tightly regulated by post‐translational modifications, particularly ubiquitylation [10, 11]. The ubiquitin‐proteasome system controls protein stability, localization, signaling, and protein‐protein interactions within these condensed phases [12, 13]. While ubiquitin conjugation can target proteins for various processes such as proteasomal degradation, immune signaling or autophagy, deubiquitylating enzymes (DUBs) reverse these modifications to stabilize proteins and modulate signaling cascades [14]. Many viruses exploit enzymes of ubiquitylation and ubiquitin‐like modifications and their corresponding hydrolases to enhance replication, transmission and immune evasion [15, 16], with several known examples of viral proteins encoding DUB activities or recruiting cellular DUBs to modify host factors in favour of virus dissemination [17, 18].

Ubiquitin C‐terminal hydrolase L3 (UCHL3) is a cysteine protease that belongs to the ubiquitin C‐terminal hydrolase (UCH) family of deubiquitylating enzymes [19]. UCHL3 cleaves ubiquitin from small adducts and processes polyubiquitin chains, thereby regulating protein stability and cellular signaling. While UCHL3 has been implicated in various cellular processes including DNA repair [20], cell cycle progression [21], and stress responses [22], its role in viral infection remains poorly described.

Despite growing appreciation for the importance of sfRNA condensates in flavivirus biology, the molecular mechanisms governing their assembly, maintenance, and regulation remain poorly understood. The post‐translational control of condensate‐associated proteins represents a largely unexplored regulatory layer that could significantly impact on immune evasion, viral persistence and spread. Given the central role of ubiquitylation in controlling protein fate and the documented ability of viruses to hijack deubiquitylating enzymes, we hypothesised that cellular DUBs might regulate sfRNA condensate dynamics to promote flavivirus replication.

Here, we perform activity‐based protein profiling to identify UCHL3 as specifically activated during infection. We investigated the role of UCHL3 in flavivirus infection and demonstrate that this DUB functions as a key pro‐viral factor. We show that UCHL3 is induced upon infection, partially translocates to the nucleus, and also associates with biomolecular condensates to retain sfRNA within them. Loss of UCHL3 results in relocalization of sfRNA away from P‐bodies, to PABPC1‐positive RNase L‐induced bodies (RLBs), resulting in degradation of viral genomic RNA. Our findings therefore reveal that UCHL3 attenuates sfRNA redistribution into antiviral RLBs, maintaining sfRNA in protective P‐body compartments and thereby promoting viral genome stability. This work reveals an unexpected intersection between the ubiquitin system and viral RNA biology, identifying UCHL3 as a regulator of sfRNA fate and a potential therapeutic target for flavivirus infections.

## 2 Results

### 2.1 UCHL3 is Activated During Viral Infection and Required for Flavivirus Production and Spread

**FIGURE 1:**
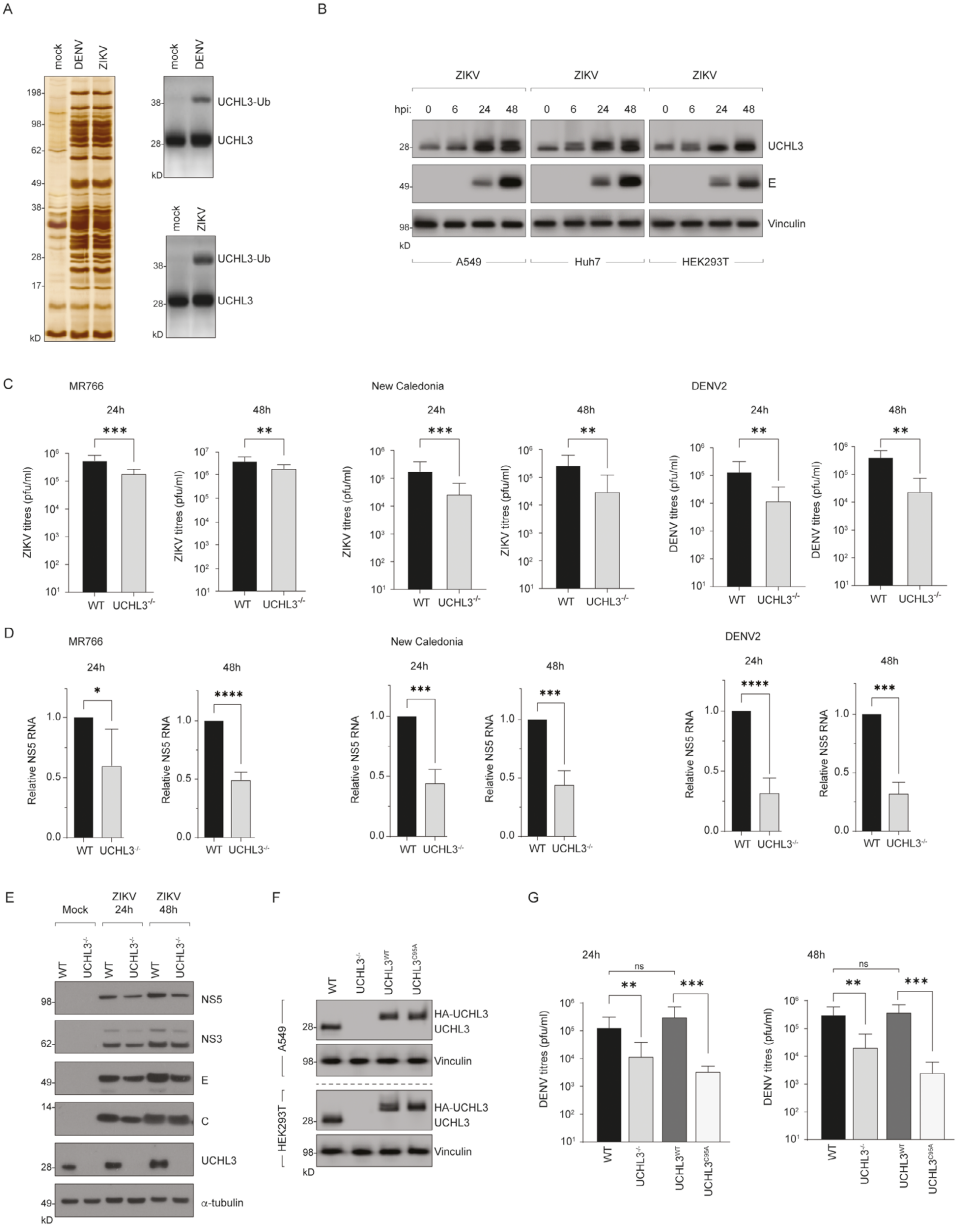
UCHL3 is activated during viral infection and supports flavivirus production. (A) Activity‐based protein profiling using ubiquitin‐vinyl methyl ester (Ub‐VME) to capture deubiquitylating enzymes activated during viral infection. *Left panel*: silver stain of total protein profiles captured; *right panel*: immunoblot detection of UCHL3 and (UCHL3‐Ub) in mock‐infected, dengue virus (DENV)‐, and Zika virus (ZIKV)‐infected cells. (B) Immunoblot analysis in A549, Huh7 and HEK293T cell lines of UCHL3 upregulation at 6, 24, and 48 h post‐infection (hpi) compared to uninfected controls (0 hpi). Viral envelope (E) protein and vinculin (loading control) are shown. (C) Viral titers (pfu/ml) in wild‐type (WT) and UCHL3^−/−^ A549 cells infected with ZIKV MR766 (African lineage), ZIKV New Caledonia (Asian lineage) or DENV2 (16681) at 24 and 48 h post‐infection. Data represent mean ± SD from triplicate experiments. **P < 0.01, ***P < 0.001. (D) Reduced viral RNA accumulation in UCHL3^−/−^ cells. Quantitative RT‐PCR analysis of NS5 RNA levels normalized to β‐actin in WT and UCHL3^−/−^ cells at 24 and 48 h post‐infection. Data represent mean ± SD. *P < 0.05, ****P < 0.0001. (E) Immunoblot analysis of viral non‐structural proteins (NS5, NS3), structural proteins (E, C), and UCHL3 in WT and UCHL3^−/−^ A549 cells under mock infection or ZIKV (MR766) infection conditions at 24 and 48 h post‐infection. α‐tubulin serves as loading control. (F) Immunoblot analysis confirming expression of reconstituted HA‐tagged wild‐type UCHL3 (UCHL3^WT^) and catalytically inactive UCHL3 (UCHL3^C95A^) in UCHL3^−/−^ A549 cells (upper) and HEK293T cells (lower) following lentiviral transduction and selection. (G) Viral titers (pfu/ml) in WT, UCHL3^–/–^, and reconstituted with wild‐type Flag‐HA‐UCHL3 (UCHL3^WT^ rescue), and catalytically inactive C95A mutant (UCHL3^C95A^). Data represent mean ± SD from triplicate experiments performed across two independent UCHL3^−/−^ clonal lines. **P < 0.01, ***P < 0.001; ns, not significant. Statistical significance determined by two‐way ANOVA with Dunnett's multiple comparisons test.

To identify deubiquitylases (DUBs) that are activated during viral infection, we employed activity‐based protein profiling using the ubiquitin‐vinyl methyl ester (Ub‐VME) probe as we reported previously [17], which covalently labels catalytically active DUBs (Figure 1A). Following infection with influenza [17], ZIKV and DENV, we observed activation of several DUBs, including USP7, USP14, OTUB1, and UCHL3, a cytoplasmic deubiquitylase of the ubiquitin C‐terminal hydrolase family (Figure 1A; Table S1). We selected UCHL3 for detailed functional characterization in this study since it displayed consistent activation across both ZIKV and DENV infections and has predicted RNA‐binding capacity. Unlike USP7 and USP14, which have well‐characterized roles in protein quality control and proteasome‐associated deubiquitylation, UCHL3's cellular functions remain less well‐defined. Our previous work characterized OTUB1 and USP25 [18] during virus infection [17]. To validate and quantify this activation, we first measured UCHL3 protein levels over infection time course. Immunoblot analyses demonstrated that UCHL3 undergoes upregulation in response to ZIKV infection, with protein levels increasing substantially above basal expression in A549, Huh7 and HEK293T cells (Figure 1B).

To investigate the functional significance of UCHL3 in flavivirus infection, we generated UCHL3 knockout (KO) A549 and HEK293T cells using CRISPR‐Cas9 gene editing (Figure S1A) and infected them with either ZIKV (African and Asian lineages) or DENV (serotype 2). UCHL3 deletion significantly reduced replication of both flaviviruses, with a more significant reduction of viral titers in dengue, both 24 and 48 h post‐infection compared to wild‐type cells (Figure 1C). This reduction in viral output was accompanied by a corresponding decrease in viral RNA accumulation, as measured by NS5 RNA levels, which showed progressive loss over time in UCHL3 KO cells infected with either virus (Figure 1D). In contrast, the impact of UCHL3 deficiency on influenza virus replication was opposite to the flavivirus phenotype where UCHL3^−/−^ cells displayed an increase in influenza polymerase activity, with no significant impact on infectious virus production (Figure S1B,C). The impaired replication in UCHL3‐deficient cells was further confirmed by analysis of viral protein expression. Western blot analysis revealed a modest reduction in steady state levels of multiple viral proteins, including the non‐structural proteins NS5 and NS3, as well as the structural protein E, in UCHL3 KO cells compared to wild‐type controls (Figure 1E). This broad reduction in viral protein accumulation across both flaviviruses suggests that UCHL3 affects either viral RNA translation or replication rather than targeting specific viral proteins.

To confirm that the observed defect in DENV/ZIKV titers was specific to UCHL3‐deficiency, we generated stable reconstituted lines. Wild‐type HA‐UCHL3 or a catalytically inactive HA‐UCHL3 variant (C95A) was reintroduced into UCHL3^−/−^ cells by lentivirus transduction, followed by selection and validation of UCHL3 expression (Figure 1F). Reconstitution with wild‐type UCHL3 rescued viral titers comparable to parental wild‐type cells (Figure 1G; Figure S1D). In contrast, expression of the catalytically dead UCHL3^C95A^ failed to rescue this defect (Figure 1G; Figure S1D), indicating that the enzymatic activity of UCHL3 is necessary for its proviral function.

### 2.2 UCHL3 Undergoes Subcellular Redistribution Upon Infection, Facilitating Flavivirus Propagation

**FIGURE 2:**
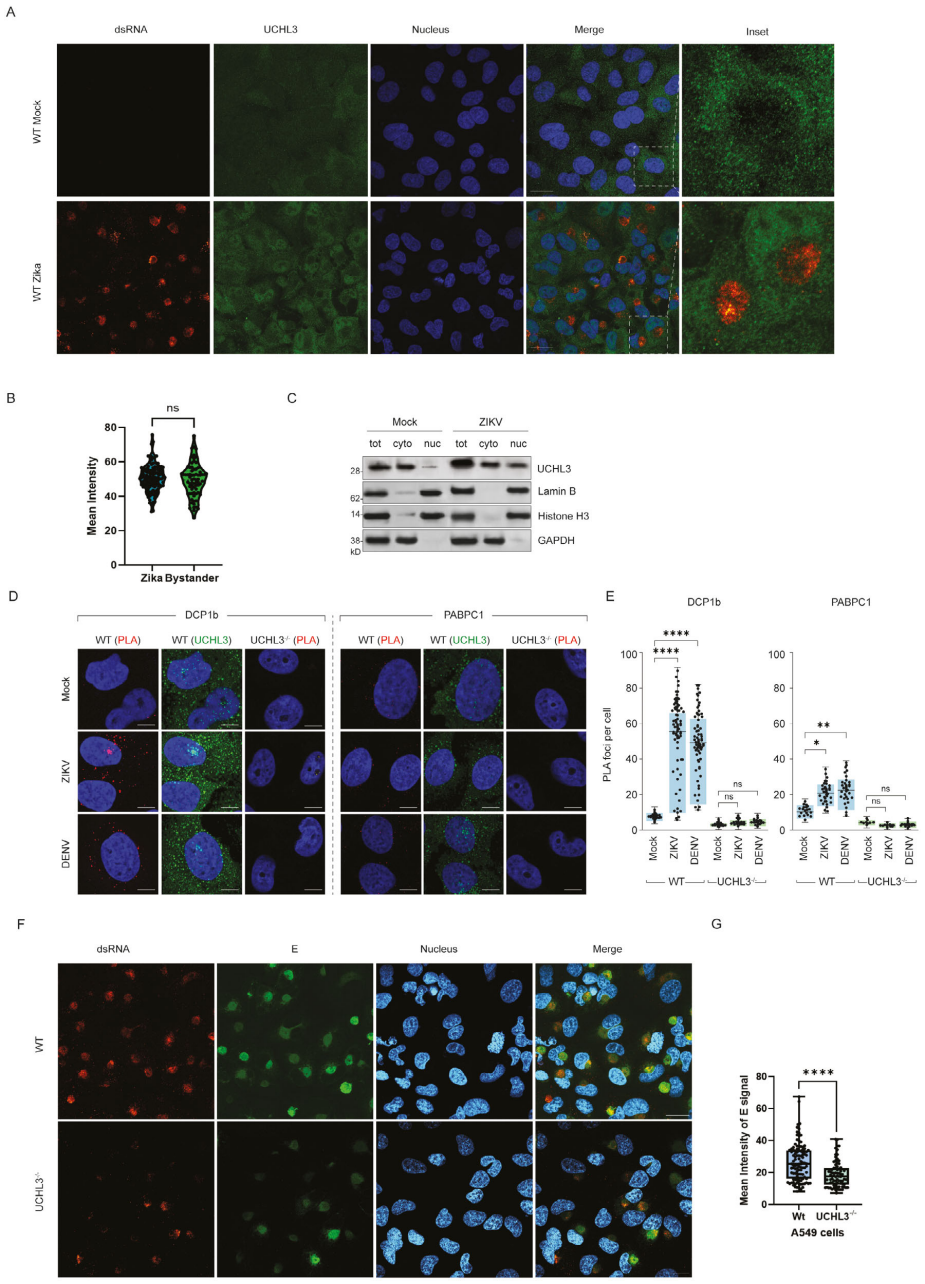
UCHL3 undergoes subcellular redistribution upon infection and associates with P‐body components. (A) Confocal microscopy images of WT A549 cells under mock infection (upper panel) and ZIKV infection at 24 h post‐infection (lower panel) and stained for double‐stranded viral RNA (dsRNA, red) to mark replication intermediates, UCHL3 (green), and nuclei (DAPI, blue). Merged images demonstrate UCHL3 redistribution from predominantly cytoplasmic localization in mock‐infected cells to dual cytoplasmic‐nuclear distribution during infection. Inset panels provide higher magnification views of individual cells. Scale bar represents 20 µm. (B) Quantitative analysis of UCHL3 nuclear accumulation in infected versus bystander cell populations. Violin plot shows mean fluorescence intensity measurements for both productively infected (ZIKV) and neighboring uninfected (Bystander) cells. ns, not significant. (C) Biochemical validation of UCHL3 subcellular redistribution. Cell fractionation followed by immunoblot analysis of total cellular lysates (tot), cytoplasmic (cyto), and nuclear (nuc) fractions from mock‐infected and ZIKV‐infected A549 cells. Lamin B (nuclear marker), Histone H3 (nuclear marker), and GAPDH (cytoplasmic marker) serve as fractionation controls. (D) Proximity ligation assay (PLA) analysis of UCHL3 interactions with condensate‐associated proteins. Representative confocal images of PLA between UCHL3 and DCP1b (P‐body marker; left panels) or PABPC1 (RLB marker; right panels) in WT and UCHL3^−/−^ A549 cells under mock, ZIKV (New Caledonia), or DENV2 (16681) infection at 24 h post‐infection. PLA signal is shown in red; UCHL3 in green. UCHL3^−/−^ cells serve as a negative control to confirm specificity of PLA signals. Nuclei are stained with DAPI (blue). Scale bar represents 10 µm. (E) Quantification of PLA foci per cell for UCHL3–DCP1b (left) and UCHL3–PABPC1 (right) interactions. Box‐and‐whisker plots with individual data points show median (center line), interquartile range (box), and whiskers extending to 1.5× IQR. Each data point represents a single cell. Data represent measurements from ≥50 cells per condition across 3 independent experiments. ****P < 0.0001, **P < 0.01, *P < 0.05; ns, not significant. (F) Immunofluorescence analysis of WT and UCHL3^−/−^ A549 cells infected with ZIKV. dsRNA (red) and viral envelope protein E (green) mark replication and assembly sites, respectively. UCHL3‐deficient cells exhibit diminished and disorganized viral compartments compared to structured perinuclear replication organelles in WT cells. Scale bar represents 20 µm. (G) Box‐and‐whisker plots with individual data points showing mean fluorescence intensity of E protein signal in WT versus UCHL3^−/−^ A549 cells. Data represent measurements from multiple fields across 3 independent experiments. ****P < 0.0001.

To characterize UCHL3 in virus infected cells, we performed subcellular localization analyses in mock and infected cells. Specificity of UCHL3 staining was validated in wild‐type and UCHL3^−/−^ cells (Figure S2). Immunofluorescence analyses revealed striking changes in UCHL3 distribution following viral infection. In mock‐infected cells, UCHL3 displayed predominantly cytoplasmic localization. However, ZIKV infection triggered substantial dual localization between the nucleus and cytoplasm (Figure 2A). Quantitative analysis confirmed that this dual‐compartment localization occurred in both productively infected cells and the bystander population (defined as cells lacking detectable dsRNA) (Figure 2B). Biochemical fractionation studies corroborated the microscopy findings, indicating clear redistribution of UCHL3 between cytoplasmic and nuclear compartments during infection (Figure 2C). The fact that both infected and bystander cells displayed this response suggests that UCHL3 relocalization is triggered by soluble inflammatory mediators or cellular stress responses rather than requiring direct interaction with viral proteins. Importantly, UCHL3 did not colocalize with viral double‐stranded RNA (dsRNA) replication intermediates, suggesting that it does not directly associate with viral replication complexes.

To determine whether UCHL3 interacts with RBPs at specific condensate compartments, we performed proximity ligation assays (PLA) between UCHL3 and DCP1b (P‐body marker) or PABPC1 (RLB marker) in mock or virus‐infected wild‐type cells (Figure 2D). PLA between UCHL3 and DCP1b revealed substantially increased punctae in virus‐infected cells compared to mock controls. On the other hand, PLA between UCHL3 and PABPC1 was only modestly increased in infected cells compared to mock controls (Figure 2D,E).

To assess the functional consequences of UCHL3 deficiency on virus‐induced ER remodeling, we examined the organization of viral replication and assembly sites using dsRNA and E protein as markers. In wild‐type cells, these structures typically manifest as distinct perinuclear compartments representing remodeled ER which house replication organelles, assembly sites, and seed LC3‐positive transport vesicles [23, 24, 25, 26]. Consistent with the observed reduction in viral titers and RNA levels, UCHL3‐deficient cells exhibited significant disruption of these virus‐induced compartments (Figure 2F), with marked reduction in the intensity of viral protein and dsRNA accumulation (Figure 2G). This architectural defect suggests that UCHL3 contributes to establishing or maintaining productive viral replication/assembly environments.

These findings collectively demonstrate that UCHL3 functions as a pro‐viral host factor that undergoes selective activation and relocalization during flavivirus infection. The infection‐induced nuclear accumulation of this normally cytoplasmic deubiquitylase, combined with its conserved requirement across multiple flaviviruses, suggests that UCHL3 serves distinct but complementary functions in different cellular compartments during the viral life cycle. While UCHL3 displays infection‐dependent proximity to condensate‐associated proteins (preferentially with P‐body components than RLB markers), its predominant diffuse cytoplasmic distribution indicates that it does not stably partition into visible RNA granules, and its regulatory effects may instead be via transient interactions or through the soluble cytoplasmic pool of RBPs.

### 2.3 UCHL3 Associates with Flaviviral sfRNA

To assess the role of UCHL3 in virus infection, we overexpressed ectopic Flag‐HA‐UCHL3 (Figure S3A). Immunoblotting analyses confirmed expression of the Flag‐HA‐UCHL3 construct (Figure S3A). Overexpression of UCHL3 resulted in increased levels of viral E and NS5 proteins. Quantitative assessment of viral replication revealed that ectopic UCHL3 expression modestly enhanced ZIKV propagation in a time‐dependent manner (Figure S3B). At 24 h post‐infection, UCHL3 overexpression resulted in a modest but measurable increase in viral titers. By 48 h post‐infection this effect was more pronounced.

**FIGURE 3:**
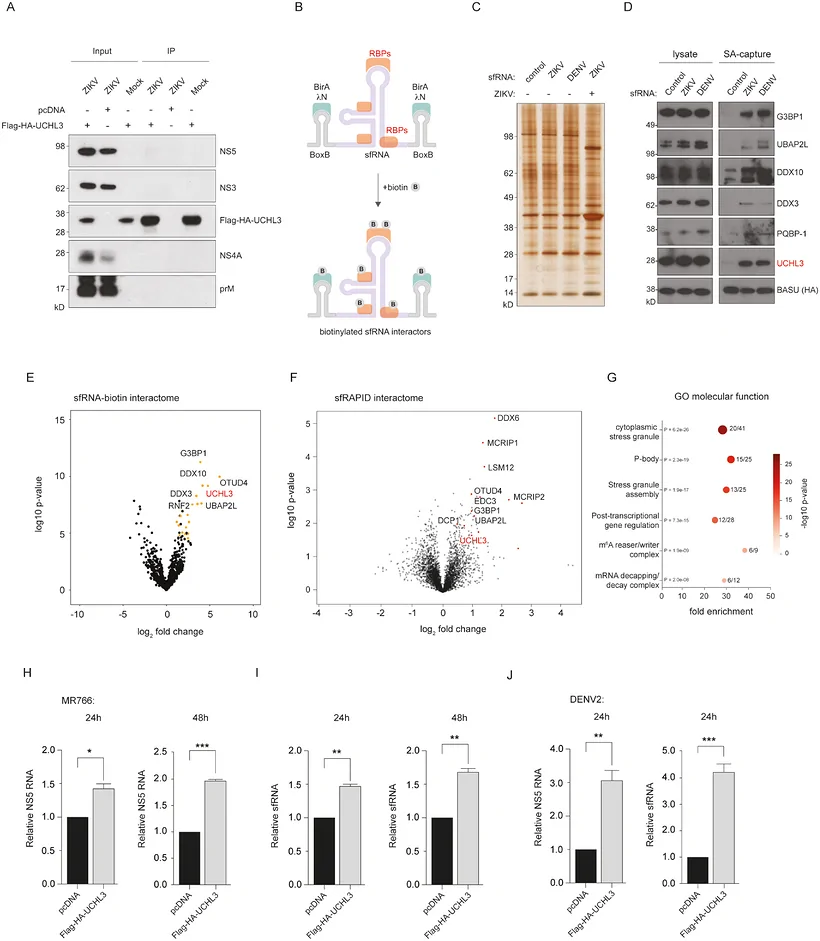
UCHL3 associates with flaviviral sfRNA. (A) Co‐immunoprecipitation of Flag‐HA‐UCHL3 from HEK293 cells transfected with either empty vector control (pcDNA3.1) or Flag‐HA‐UCHL3 construct and infected with ZIKV or mock‐infected. Input lysates and immunoprecipitated (IP) fractions were analyzed by immunoblotting for viral non‐structural proteins NS5, NS3, and NS4A, viral structural protein prM, and Flag‐HA‐UCHL3. (B) Schematic representation of the biotinylated sfRNA‐interactome capture strategy (sfRAPID). The engineered sfRNA construct contains flanking BoxB stem‐loop structures that specifically recruit the BirA‐λN fusion protein (BASU). Upon biotin supplementation, BASU biotinylates proteins in close proximity to sfRNA, enabling streptavidin‐mediated affinity purification of sfRNA‐associated ribonucleoprotein complexes. (C) Silver staining analysis of sfRNA‐interacting protein complexes. Total protein profiles from streptavidin captures using control RNA motif, ZIKV sfRNA, or DENV sfRNA constructs ± ZIKV infection as indicated, demonstrating infection‐dependent recruitment of cellular factors to sfRNA assemblies. (D) Streptavidin‐affinity capture followed by immunoblot analysis. Cell lysates and streptavidin‐captured (SA‐capture) fractions from cells expressing control, ZIKV sfRNA, or DENV sfRNA constructs were probed for G3BP1, UBAP2L, DDX10, DDX3, PQBP‐1, UCHL3 (red), and BASU (HA‐tagged, internal control). Selective enrichment of condensate‐associated RNA‐binding proteins and UCHL3 in the SA‐capture fraction confirms their association with sfRNA‐containing ribonucleoprotein complexes. (E) Volcano plot of the sfRNA‐biotin interactome (direct biotinylated sfRNA pulldown followed by LC–MS/MS). Significantly enriched RNA‐binding candidates (highlighted in orange; e.g., G3BP1, DDX10, DDX3, RNF2, UBAP2L, OTUD4, UCHL3) are shown relative to control RNA. Axes show log_2_ fold change (x) and −log_10_ p‐value (y). (F) Volcano plot of the sfRAPID proximity‐labeling interactome. Proteins enriched in sfRNA proximity include P‐body and stress granule components (DDX6, LSM12, MCRIP1, MCRIP2, OTUD4, EDC3, G3BP1, DCP1, UBAP2L). (G) Gene Ontology (GO) molecular function enrichment analysis of the sfRAPID interactome. Bubble plot shows significantly enriched GO categories, with bubble size proportional to gene count, colour reflecting −log_10_ p‐value, and x‐axis indicating fold enrichment. Top categories include cytoplasmic stress granule, P‐body, stress granule assembly, post‐transcriptional gene regulation, m^6^A reader/writer complex, and mRNA decapping/decay complex. (H) Quantitative RT‐PCR analysis of NS5 genomic RNA levels in HEK293 cells transfected with either pcDNA3.1 empty vector or Flag‐HA‐UCHL3 at 24 and 48 h post‐ZIKV infection. Data represent mean ± SD from triplicate experiments. *P < 0.05, ***P < 0.001. (I) Quantitative RT‐PCR analysis of sfRNA levels under the same conditions as (H). Ectopic UCHL3 expression increases sfRNA accumulation at both 24 and 48 h post‐infection. Data represent mean ± SD from triplicate experiments. **P < 0.01. (J) Quantitative RT‐PCR analysis of NS5 genomic RNA (left) and sfRNA (right) levels in HEK293 cells transfected with pcDNA3.1 or Flag‐HA‐UCHL3 at 24 h post‐DENV2 (16681) infection. Ectopic UCHL3 expression enhances accumulation of both RNA species during DENV infection. Data represent mean ± SD from triplicate experiments. **P < 0.01, ***P < 0.001.

To test whether UCHL3 directly engages viral components, we first performed co‐immunoprecipitation of Flag‐HA‐UCHL3 from transfected 293 cells, both mock and ZIKV‐infected. Viral non‐structural (NS5, NS3, NS4A) and structural (prM) proteins did not co‐precipitate with UCHL3, arguing against a stable UCHL3‐viral protein interaction (Figure 3A). Given the observation that UCHL3 deficiency affects viral RNA accumulation, and its predicted interaction with RNA, we hypothesized that UCHL3 might instead associate with viral RNA‐protein complexes rather than individual viral proteins. UCHL3's known interactors include several RNA‐binding proteins (BioGRID), and its paralogue UCHL5 has been identified as a multifunctional RNA‐binding protein influencing both mRNA abundance and translational efficiency [27].

To systematically identify proteins within the sfRNA ribonucleoprotein neighborhood, we adapted a proximity‐biotinylation sfRNA‐interactome capture strategy [28] (sfRAPID; Figure 3B). This approach was selected because it captures transient and indirect RNA‐protein interactions that may be missed by traditional co‐immunoprecipitation, identifies the protein neighborhood surrounding sfRNA without requiring direct UCHL3‐sfRNA binding, and enables unbiased discovery of sfRNA‐associated factors. Silver staining analysis of biotinylated sfRNA‐interactors captured on streptavidin beads revealed distinct protein profiles between control and ZIKV‐infected conditions (Figure 3C), indicating infection‐dependent recruitment of cellular factors to sfRNA complexes. To validate that ectopically expressed sfRNA‐BoxB constructs recapitulate the subcellular behavior of endogenous sfRNA, we performed RNA‐FISH comparing the localization of plasmid‐derived sfRNA with that of endogenous sfRNA detected using virus‐specific probes during authentic ZIKV infection. Both ectopic and endogenous sfRNA species showed comparable cytoplasmic punctate distribution and colocalization with P‐body markers (Figure S3C), confirming that the sfRAPID system faithfully reports on sfRNA‐associated protein interactions.

Streptavidin‐affinity capture coupled with immunoblot analysis demonstrated that UCHL3 is specifically recruited to sfRNA ribonucleoprotein complexes (Figure 3D). In the presence of sfRNA from either ZIKV or DENV virus, UCHL3 was efficiently captured in the streptavidin pulldown fraction, while control conditions with scrambled sfRNA showed minimal UCHL3 recovery. Importantly, this sfRNA‐mediated recruitment of UCHL3 was accompanied by the co‐purification of G3BP1, a well‐characterized stress granule component known to associate with sfRNA. The parallel enrichment of UCHL3, G3BP1, and additional RNA‐binding proteins associated with biomolecular condensates (DDX10, DDX3, UBAP2L, PQBP1) alongside the biotinylated internal control (BASU) in the pulldown fraction indicates that UCHL3 interacts with sfRNA‐containing ribonucleoprotein complexes, although whether this association occurs within assembled condensates or through the soluble cytoplasmic pool of these proteins remains to be determined. Unbiased LC‐MS/MS using biotinylated sfRNA or sfRAPID provided orthogonal confirmation of enrichment of cytoplasmic RNA‐granule scaffolds and enzymes including G3BP1, DDX3, DDX6, UBAP2L, and OTUD4, relative to control RNA (Figure 3E–G).

To assess the functional consequences of UCHL3's association with viral RNA complexes, we performed quantitative RT‐PCR analysis of viral RNA levels in cells expressing UCHL3 (Figure 3H,J). Ectopic expression of Flag‐HA‐UCHL3 resulted in significant increase of NS5 genomic RNA accumulation at 24 and 48 h post‐infection (Figure 3H,J). Similarly, sfRNA levels were substantially elevated in UCHL3‐overexpressing cells (Figure 3I,J). These data indicate that UCHL3 not only physically associates with viral RNA‐protein complexes but also functionally promotes the accumulation of both genomic and subgenomic viral RNA species. Collectively, these findings suggest that UCHL3 might function to regulate sfRNA stability and accumulation during flavivirus infection. While the fold‐changes with UCHL3 overexpression are modest (particularly with ZIKV), this likely reflects saturation of endogenous substrates. Dose‐response experiments with varying amounts of transfected UCHL3 confirmed a concentration‐dependent relationship between UCHL3 levels and sfRNA accumulation (Figure S3D,E).

### 2.4 UCHL3 Deficiency Triggers RNase L Activation and sfRNA Sequestration

**FIGURE 4:**
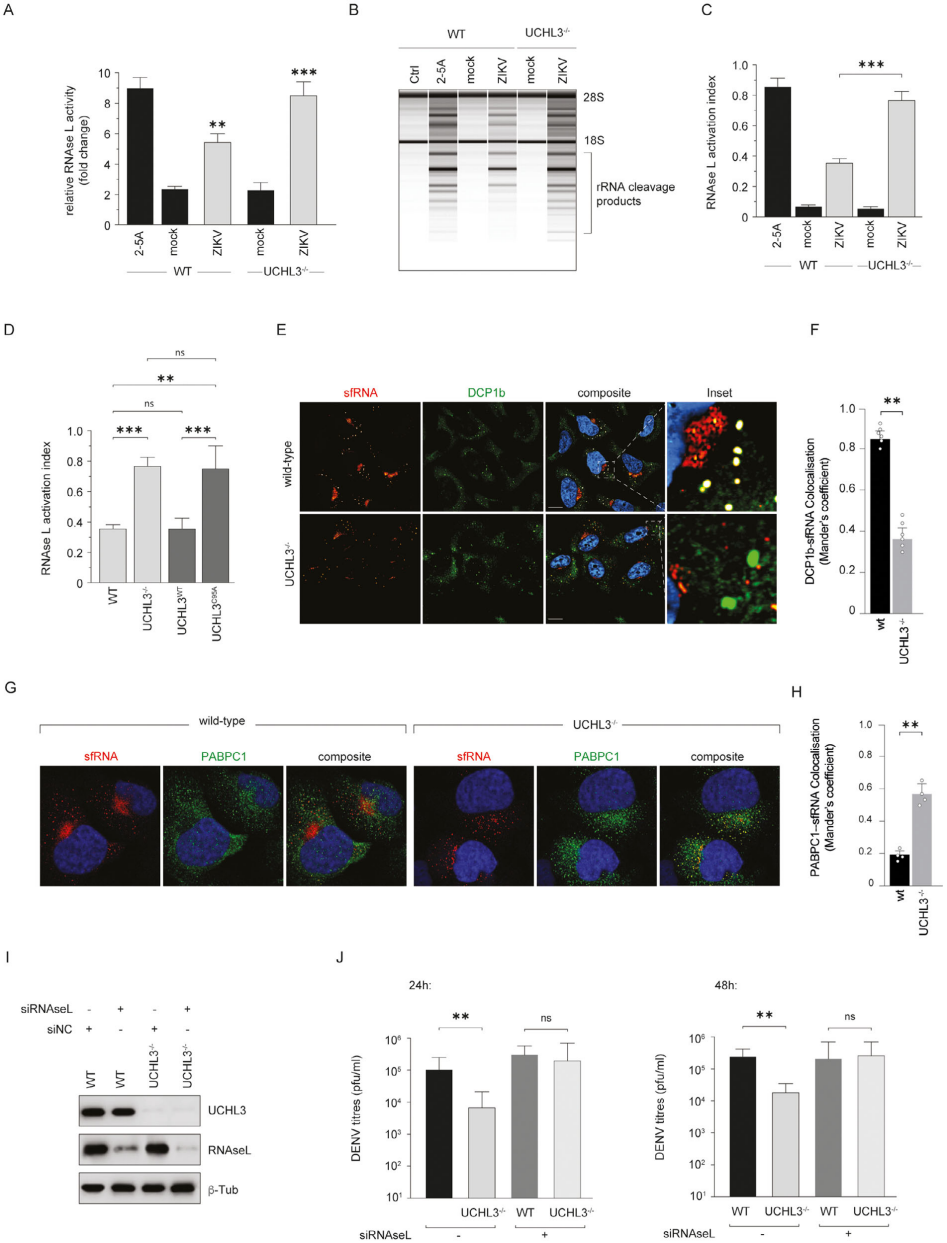
UCHL3 deficiency triggers RNase L activation and sfRNA sequestration. (A) Quantitative analysis of RNase L activation in WT and UCHL3^−/−^ A549 cells during ZIKV infection, measured by FRET‐based 2–5A assay. 2–5A treatment serves as a positive control for RNase L pathway activation. Data represent mean ± SD from triplicate experiments. **P < 0.01, ***P < 0.001. (B) Electrophoretic analysis (Bioanalyzer) of total RNA extracted from WT and UCHL3^−/−^ A549 cells under control (Ctrl), 2–5A treatment, mock infection, or ZIKV infection conditions. (C) Quantification of rRNA integrity from (B), expressed as an RNase L activation index (ratio of cleaved to total rRNA species). Data represent mean ± SD from triplicate experiments. ***P < 0.001. (D) RNase L activation index in WT, UCHL3^−/−^, UCHL3^WT^ rescue, and UCHL3^C95A^ cells during ZIKV infection. Data represent mean ± SD from triplicate experiments. **P < 0.01, ***P < 0.001; ns, not significant. (E) Fluorescence in situ hybridization (FISH) coupled with immunofluorescence microscopy reveals sfRNA subcellular distribution (red) relative to P‐body marker DCP1b (green) in WT and UCHL3^−/−^ cells infected with ZIKV at 24 h post‐infection. Composite images show DAPI‐stained nuclei (blue). Inset panels provide high‐magnification views of sfRNA‐containing structures. Scale bar represents 10 µm. (F) Quantitative colocalization analysis of DCP1b‐sfRNA association. Manders' coefficient measurements of sfRNA retention within P‐body compartments in UCHL3^−/−^ cells compared to WT controls. Data represent measurements from ≥50 cells per condition across 3 independent experiments. **P < 0.01. (G) sfRNA‐FISH combined with PABPC1 immunofluorescence in WT and UCHL3^−/−^ cells infected with ZIKV at 24 h post‐infection. sfRNA (red), PABPC1 (green), and DAPI (blue) are shown with composite overlays. Scale bar represents 10 µm. (H) Quantitative colocalization analysis of PABPC1‐sfRNA association. Manders' coefficient measurements of sfRNA–PABPC1 colocalization in UCHL3^−/−^ cells compared to WT controls. Data represent measurements from ≥50 cells per condition across 3 independent experiments. **P < 0.01. (I) Immunoblot analysis confirming depletion of RNase L protein levels following siRNA treatment (siRNase L) compared to non‐targeting control (siNC) in both WT and UCHL3^−/−^ A549 cells. β‐tubulin serves as loading control. (J) DENV2 viral titers (pfu/ml) in WT and UCHL3^−/−^ cells treated with control siRNA (siNC) or RNase L‐targeting siRNA (siRNase L) at 24 and 48 h post‐infection. Data represent mean ± SD from triplicate experiments. **P < 0.01; ns, not significant.

To elucidate the molecular mechanism by which UCHL3 depletion impairs flavivirus replication, we investigated its impact on the 2’‐5’ oligoadenylate synthetase/RNase L (OAS/RNase L), a key innate immune effector system activated by viral dsRNA. To quantify RNase L activation, we employed a fluorogenic FRET‐based assay in which cell lysates are incubated with recombinant human RNase L and a dual‐labelled RNA substrate. Endogenous 2–5A present in the lysate activates RNase L, which cleaves the reporter RNA and generates fluorescence proportional to 2–5A concentration. Accumulation of endogenous 2–5A in UCHL3^−/−^ cells was substantially higher compared to wild‐type controls during ZIKV infection (Figure 4A). This enhanced 2–5A synthesis was accompanied by pronounced ribosomal RNA (rRNA) degradation, as evidenced by electrophoretic analysis demonstrating characteristic rRNA cleavage products in UCHL3^−/−^ cells (Figure 4B). We calculated a RNase L activation index previously defined as the ratio of cleaved rRNA species to intact (28S + 18S) rRNA [29]. This analysis revealed a significantly elevated RNase L activation index in UCHL3^−/−^ cells compared to wild‐type cells during ZIKV infection (Figure 4C). These data were further corroborated by Bioanalyzer analysis of rRNA integrity at matched timepoints (Figure S4A,B). Importantly, reconstitution of UCHL3^−/−^ cells with UCHL3^WT^ restored 2–5A levels and rRNA integrity to near wild‐type levels, whereas the catalytically inactive C95A mutant failed to suppress RNase L hyperactivation, confirming that UCHL3 enzymatic activity is required to restrain the OAS/RNase L pathway during infection (Figure 4D).

To resolve the temporal dynamics of RNase L activation, we performed Bioanalyzer analysis of rRNA integrity at 6, 12, 18 and 24 h post‐infection in wild‐type and UCHL3^–/–^ cells (Figure S4A,B). In wild‐type cells, rRNA cleavage products first became detectable at ∼18 h post‐infection, consistent with previous reports that approximately 70% of A549 cells activate RNase L by 24 h during ZIKV infection [9]. In UCHL3^–/–^ cells, rRNA cleavage was detected earlier (∼12 h post‐infection) and accumulated to a greater extent at later time points, indicating that UCHL3 deficiency both accelerates the onset and increases the magnitude of RNase L activation. Together, these data demonstrate that UCHL3 deficiency accelerates the kinetics of RNase L activation during flavivirus infection, with a greater fraction of cells engaging the antiviral response at earlier time points.

To visualize the spatial consequences of enhanced RNase L activity on viral RNA localization, we employed fluorescence in situ hybridization (FISH) coupled with immunofluorescence microscopy to track sfRNA subcellular distribution. We note that RNase L activation occurs in a substantial fraction of wild‐type A549 cells during ZIKV infection, with sfRNA partitioning to both P‐bodies and RLBs in unmanipulated cells [9]. We hypothesized that UCHL3‐deficiency shifts the sfRNA population partitioning between these compartments. In wild‐type cells, sfRNA exhibited characteristic colocalization with P‐body markers (DCP1b), consistent with its incorporation into pro‐viral ribonucleoprotein condensates (Figure 4E,F). However, in UCHL3^−/−^, sfRNA underwent relocalization away from DCP1b‐positive P‐bodies, instead appearing in distinct cytoplasmic structures.

To determine whether these structures represent bona fide RNase L‐induced bodies (RLBs), we performed sfRNA‐FISH combined with immunofluorescence for PABPC1, an established RLB marker that is enriched in RLBs but absent from P‐bodies [30, 31]. In wild‐type cells, sfRNA predominantly colocalized with DCP1b‐positive P‐bodies and showed minimal PABPC1 overlap. In contrast in UCHL3^–/–^ cells, the sfRNA‐containing foci showed significant enrichment for PABPC1 (Figure 4G), consistent with their identity as RLBs. Colocalization analysis demonstrated a significant increase in sfRNA‐PABPC1 colocalization (Figure 4H), indicating that UCHL3 is essential for maintaining sfRNA within protective pro‐viral compartments.

To establish that sfRNA redistribution to PABPC1‐positive foci in UCHL3‐deficient cells is dependent on RNase L, we examined sfRNA localization in RNase L‐knockdown cells and in UCHL3^–/–^/RNase L double‐deficient cells (Figure 4I; Figure S4C,D). In RNase L‐knockdown A549 cells infected with ZIKV, sfRNA maintained its association with DCP1b‐positive P‐bodies, and the PABPC1‐positive cytoplasmic foci observed in UCHL3^–/–^ cells were largely absent, confirming that formation of these structures requires RNase L. Critically, in UCHL3^–/–^/RNase L double‐deficient cells, sfRNA redistribution away from P‐bodies was substantially reversed: sfRNA‐DCP1b colocalization was restored to near‐wild‐type levels, and PABPC1‐positive foci were not detected. These data demonstrate that the sfRNA relocalization phenotype observed in UCHL3^–/–^ cells is RNase L‐dependent, and that the cytoplasmic structures to which sfRNA redistributes in the absence of UCHL3 are bona fide RNase L‐induced bodies characterized by PABPC1 enrichment.

To establish the causal relationship between RNase L hyperactivation and the observed phenotypes, we performed rescue experiments using RNase L knockdown in the wild‐type and UCHL3‐deficient background (Figure 4I,J). Western blot analysis confirmed efficient depletion of RNase L protein levels following siRNA treatment in both wild‐type and UCHL3^−/−^ cells, whilst UCHL3 levels remained unaffected by the RNase L knockdown. At 24 h post‐infection, UCHL3^−/−^ cells showed reduced DENV titers compared to wild‐type controls. Remarkably, knockdown of RNase L in UCHL3^−/−^ cells substantially rescued viral replication, with titers similar to wild‐type cells. This rescue effect was maintained at 48 h post‐infection, demonstrating that the reduced viral titers in UCHL3 deficient cells is largely RNase L hyperactivation dependent.

These findings collectively demonstrate that UCHL3 functions as a regulator that suppresses RNase L activation during flavivirus infection, thereby enabling viral RNA to evade degradation. UCHL3 delays sfRNA redistribution to RLBs, potentially providing a temporal window during which viral replication proceeds efficiently. In the absence of UCHL3, sfRNA is sequestered into PABPC1‐positive RLBs, resulting in enhanced viral RNA decay and reduced viral persistence. These data are consistent with a model in which UCHL3 modulates the partitioning of sfRNA between pro‐viral and antiviral condensates, thereby determining the fate of viral RNA and the success of infection.

### 2.5 UCHL3 Promotes ZIKV Infection Through Interferon‐Independent Mechanisms

To investigate whether UCHL3's pro‐viral function operates through modulation of cellular antiviral responses, we examined interferon‐stimulated gene (ISG) expression under various combinations of viral infection and exogenous interferon treatment. This experimental design allows us to dissect the complex interplay between UCHL3, innate immune signaling, and viral replication, to determine whether UCHL3's effects are primarily immunomodulatory or operate through alternative mechanisms.

**FIGURE 5:**
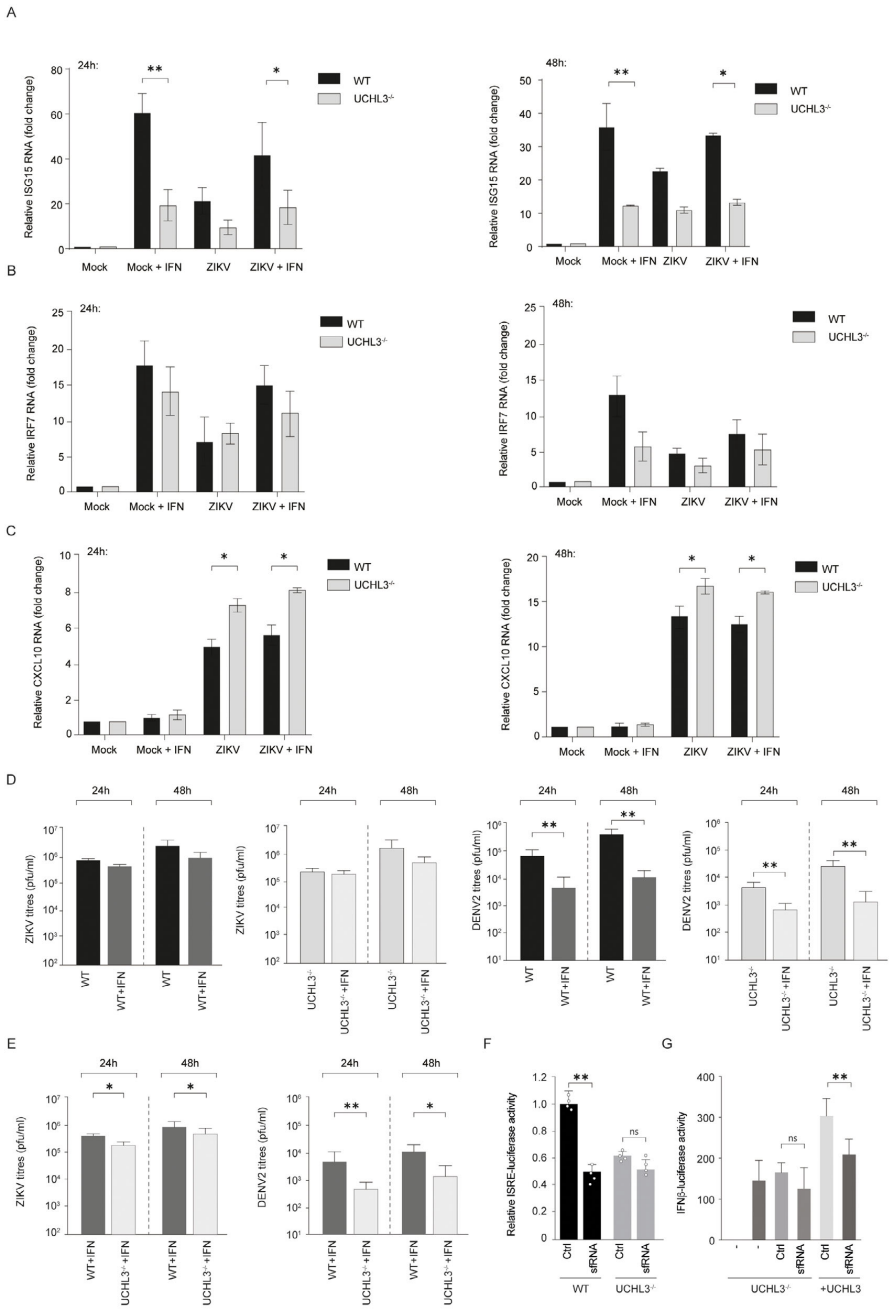
UCHL3 promotes ZIKV infection through interferon‐independent mechanisms. (A) Quantitative RT‐PCR analysis of ISG15 mRNA levels in wild‐type (WT) and UCHL3^−/−^ A549 cells under four experimental conditions: mock infection, mock infection with exogenous type‐I interferon treatment (Mock + IFN), ZIKV infection alone, and ZIKV infection with interferon treatment (ZIKV + IFN) at 24 and 48 h. Data represent mean ± SD from triplicate experiments. *P < 0.05, **P < 0.01. (B) IRF7 mRNA expression analysis under identical experimental conditions as (A). Data represent mean ± SD from triplicate experiments. (C) CXCL10 mRNA levels under identical experimental conditions as (A). UCHL3^−/−^ cells exhibit enhanced CXCL10 expression during viral infection but reduced responsiveness to interferon treatment, suggesting differential regulatory mechanisms for distinct interferon‐responsive genes. Data represent mean ± SD from triplicate experiments. *P < 0.05. (D) Viral titers (pfu/ml) in WT and UCHL3^−/−^ cells with and without exogenous interferon treatment for ZIKV (*left*) and DENV2 (*right*) at 24 and 48 h post‐infection. UCHL3^−/−^ cells maintain significantly reduced viral replication even when interferon responses are supplemented exogenously. Data represent mean ± SD from triplicate experiments. **P < 0.01. (E) Viral titers comparing interferon‐treated WT and UCHL3^−/−^ cells under identical stimulation conditions for ZIKV (*left*) and DENV2 (*right*) at 24 and 48 h post‐infection. Data represent mean ± SD from triplicate experiments. *P < 0.05, **P < 0.01. (F) ISRE‐luciferase reporter assay measuring interferon‐stimulated response element (ISRE) activity in WT and UCHL3^−/−^ cells transfected with control RNA (Ctrl) or sfRNA following exogenous type‐I interferon stimulation. Data are normalized to IFN‐stimulated control and represent mean ± SD from triplicate experiments. **P < 0.01; ns, not significant. (G) IFN‐β promoter luciferase reporter assay in UCHL3^−/−^ HEK293T cells transfected with control RNA or ZIKV sfRNA in the presence or absence of co‐expressed UCHL3, followed by poly I:C stimulation (5 µg/ml) for 24 h. Data are expressed as RLU fold induction normalized to untreated cells and represent mean ± SD from three independent experiments. **p < 0.01.

First, we analyzed ISG15 mRNA levels, a canonical marker of interferon pathway activation, in wild‐type and UCHL3^−/−^ cells under four distinct conditions: mock infection, mock infection with exogenous interferon treatment, ZIKV infection alone, and ZIKV infection followed by interferon treatment (Figure 5A). In mock‐infected conditions, both wild‐type and UCHL3^−/−^ cells displayed minimal ISG15 expression as anticipated, whereas exogenous interferon treatment resulted in a dramatic increase in ISG15 in wild‐type cells, and a blunted response in UCHL3^−/−^ cells. This indicates that UCHL3 functions as a positive regulator of interferon signaling, likely acting downstream of the interferon receptor to facilitate transcriptional responses. ZIKV infection alone resulted in suppressed ISG15 expression in both cell types, consistent with established flavivirus immune evasion mechanisms. While wild‐type cells maintained relatively low ISG15 levels, UCHL3^−/−^ cells exhibited further reduction, indicating that it helps ISG expression even under conditions of viral immune suppression. When ZIKV infection was combined with exogenous interferon treatment, we observed partial restoration of ISG15 expression in wild‐type cells, demonstrating that some interferon responsiveness is retained despite viral countermeasures. However, this rescue was significantly impaired in UCHL3^−/−^ cells, indicating that UCHL3 deficiency results in persistent refractoriness to interferon signaling, even when interferon is provided exogenously.

To determine whether this pattern extends to other ISGs, we examined IRF7 expression under identical conditions (Figure 5B). The results largely recapitulated the ISG15 findings, with UCHL3^−/−^ cells showing reduced responsiveness to interferon stimulation. Interestingly, the magnitude of this effect was somewhat less pronounced for IRF7, suggesting that different ISGs may have varying dependencies on UCHL3 for optimal expression, reflecting the complexity of interferon‐mediated transcriptional programs.

We also analyzed CXCL10, a chemokine that serves as both an interferon‐stimulated gene and an important mediator of antiviral immunity (Figure 5C). Interestingly, the pattern of CXCL10 expression revealed the opposite effect, with UCHL3^−/−^ cells showing enhanced expression during viral infection but reduced responsiveness to interferon treatment. This paradoxical increase in CXCL10 during infection in UCHL3‐deficient cells may reflect compensatory inflammatory responses or altered regulation of this particular gene in the absence of UCHL3.

To test whether this immunomodulatory function accounts for UCHL3's pro‐viral effects, we measured viral titers under conditions designed to equalize interferon responses between wild‐type and UCHL3^−/−^ cells (Figure 5D). In the absence of exogenous interferon, UCHL3^−/−^ cells showed the expected reduction in ZIKV titers compared to wild‐type controls, consistent with our previous findings. Crucially, when both cell types were treated with exogenous interferon, thus bypassing differences in endogenous interferon responsiveness, UCHL3^−/−^ cells continued to show significantly reduced viral titers compared to wild‐type cells. This finding is further reinforced by direct comparison of interferon‐treated wild‐type and UCHL3^−/−^ cells (Figure 5E). Even under conditions where both cell types are exposed to identical interferon stimulation, UCHL3^−/−^ cells supported significantly lower levels of viral replication at both time points examined. The persistence of this phenotype under interferon‐equalized conditions demonstrates that UCHL3's pro‐viral function operates through mechanisms that are fundamentally independent of its role in interferon signaling.

These data establish UCHL3 as a multifunctional protein with distinct roles in both immune signaling and viral replication. While UCHL3 functions as a positive regulator of interferon responses, its critical pro‐viral effects during flavivirus infection operate through alternative mechanisms that are independent of interferon pathway modulation, potentially via viral RNA metabolism and condensate biology as the primary basis for its role in flavivirus pathogenesis. We note that the reduced ISG expression in UCHL3‐deficient cells may also reflect RNase L‐mediated degradation of ISG mRNAs [32, 33].

To further assess whether UCHL3 modulates sfRNA's known biological activities, we examined a key functional readout, where sfRNA is known to suppress interferon responses. To test whether UCHL3 is required for this immunomodulatory activity, we employed ISRE‐luciferase reporter assays in the presence or absence of ectopically expressed sfRNA. sfRNA overexpression suppressed interferon‐stimulated reporter activity in wild‐type cells, consistent with its established immunosuppressive function. Critically, this suppression was abolished in UCHL3‐deficient cells (Figure 5F), demonstrating that UCHL3 is required for sfRNA to exert its immunomodulatory effects. To determine whether this requirement extends to the upstream induction of type‐I interferon, we performed IFN‐β promoter luciferase reporter assays in UCHL3^−^^/^^−^ cells co‐transfected with sfRNA in the presence or absence of reconstituted UCHL3, followed by poly I:C stimulation. sfRNA failed to suppress IFN‐β promoter activity in UCHL3‐deficient cells; however, co‐expression of UCHL3 restored the ability of sfRNA to significantly reduce IFN‐β promoter‐driven transcription (Figure 5G). These data demonstrate that UCHL3 is required for sfRNA‐mediated antagonism at both the interferon induction and signaling levels. Together, these data provide functional evidence that UCHL3 modulates sfRNA's biological activities, including RBP sequestration and interferon antagonism.

## 3 Discussion

Our findings establish UCHL3 as a key deubiquitylase that flaviviruses exploit to maintain viral genomic stability through control of RNA‐protein condensate dynamics. Cellular RNA metabolism occurs within membrane‐free biomolecular condensates [34, 35] including P‐bodies [36], stress granules [37], and the recently characterized RNase L‐induced bodies (RLBs) [9, 31, 38]. The structures represent competing fates for viral RNA, with dramatically different consequences for viral persistence. Our data indicate that UCHL3 modulates the kinetics of sfRNA partitioning between P‐bodies and RLBs, delaying the transition from protective to degradative compartments – a previously unrecognized layer of post‐translational control over RNA granule fate determination. Importantly, this proviral function is conserved across African and Asian lineage ZIKV strains and DENV2, although the magnitude of UCHL3 dependence differs between viruses: DENV2 shows substantially greater sensitivity to UCHL3 loss. This differential may reflect differences in sfRNA abundance relative to genomic RNA, their structures, in the potency of NS5‐mediated interferon antagonism, or in replication kinetics that alter the temporal window during which RNase L‐mediated decay can act.

A central question is how UCHL3 biases sfRNA toward P‐bodies. Several condensate scaffolds are shared between P‐bodies and RLBs including G3BP1 [31, 39] and UBAP2L [39], complicating simple models where UCHL3 stabilizes single compartments. One possibility is that UCHL3 preferentially deubiquitylates P‐body specific components not shared with RLBs, (DCP1 [40], DCP2 [40], EDC3 [40, 41], XRN1 [9]), thereby stabilizing the P‐body scaffold and retain sfRNA within them. Alternatively, UCHL3 may act upstream of condensate partitioning by restraining RNase L activation itself. These models are not mutually exclusive, and future work identifying the specific UCHL3 substrates will be required to distinguish between the two. The requirement for catalytic activity, demonstrated by the failure of the C95A mutant to rescue either viral replication or RNase L hyperactivation, implicates substrate deubiquitylation rather than passive scaffolding functions. Identifying these substrates through in vitro deubiquitylation assays and substrate trapping, together with the cognate ubiquitin ligases that carry out the opposing modification, will be essential to resolve the primary mechanism. This mechanism is well‐aligned with the growing literature demonstrating that ubiquitin signaling shapes RNA granule dynamics. As exemplified by OTUD4, in stress granule regulation [42].

It is worth noting that only a small fraction of RNA‐binding proteins partition into assembled granules, with the majority remaining in the soluble cytoplasmic pool. Our proximity ligation data indicate that UCHL3 associates with DCP1b‐containing complexes in an infection‐dependent manner but does not stably partition into visible condensates. UCHL3 may therefore exert its regulatory effects via transient interactions or by deubiquitylating RBPs prior to their incorporation into granules.

Beyond condensate regulation, sfRNA may contribute to viral replication organelle (vRO) biogenesis [43, 44]. Viral 3' UTR sequences contain cis‐acting elements that recruit host factors involved in membrane remodeling and establishing sites of viral genome amplification [23, 24, 45, 46]. sfRNA could nucleate ribonucleoprotein platforms that couple to membrane‐shaping machinery, consistent with the hijacking of IGF2BP2 during ZIKV infection [47], and with broader Flaviviridae precedents linking 3'‐UTR‐RBP interactions to ER remodeling [48].

While our data support the condensate‐centric model outlined above, alternative possibilities exist. First, UCHL3 could influence the OAS–RNase L pathway directly [49] by deubiquitylating components of this pathway, rather than condensate scaffolds. Under this model, altered sfRNA localization would be a secondary consequence. The failure of catalytically‐dead UCHL3 to rescue viral replication defect or RNase L hyperactivation is consistent with both models. Second, the reduced ISG expression observed in UCHL3‐deficient cells may reflect RNase L‐mediated suppression of antiviral gene expression through inhibition of nuclear mRNA export and transcriptional repression at immune‐related loci [32, 33]. Third, our experiments were performed in transformed cell lines, which may not fully recapitulate the physiological responses.

Post‐translational regulation of RNA fate is likely to extend beyond flavivirus infections. Recent studies have shown that a range of RNA viruses generate stable subgenomic species, including coronaviruses and togaviruses [50] and may exploit analogous mechanisms. UCHL3 or related deubiquitylases could therefore represent broadly applicable targets for RNA virus control.

## 4 Methods

### 4.1 Cell Lines

A549 (human lung adenocarcinoma; ATCC, CCL‐185), Huh7 (as previously described [23, 26, 46]), 293T (ATCC, CRL‐3216), and HEK293 (ATCC, CRL‐1573) cells were maintained in complete Dulbecco's Modified Eagle Medium (cDMEM) (Gibco, 11965092) supplemented with 10% foetal bovine serum (FBS) (Gibco, 10270106) and 20mM HEPES buffer (Sigma), at 37 °C with 10% CO_2_. Vero cells (African green monkey kidney; ATCC, CCL‐81) were maintained in DMEM supplemented with 1% FBS and 20 mM HEPES buffer, at 37°C with 10% CO_2_. Cells were passaged sub‐confluently with 0.25% Trypsin‐EDTA (Gibco, 25200056). All cell lines tested negative for mycoplasma contamination using the MycoAlert Mycoplasma Detection Kit (Lonza, LT07‐318) prior to experiments. A549 UCHL3^−/−^ and HEK293T UCHL3^−/−^ cells were generated in‐house as described below.

### 4.2 Virus Stocks

ZIKV strain MR‐766 (African lineage, 1947; GenBank accession: LC002520) was obtained from the European Virus Archive (EVAg, Ref‐SKU: 001v‐EVA143). ZIKV strain NC‐2014‐5132 (Asian lineage, New Caledonia, 2014; GenBank accession: SRR5309452) was kindly provided by Dr Myrielle Dupont‐Rouzeyrol, Institut Pasteur de Nouvelle‐Calédonie. Dengue virus serotype 2 (DENV2) strain 16681 (GenBank accession: U87411) was obtained from BEI Resources. Vero cells (1 × 10^6^) were infected at MOI 0.01 and incubated for 1 h in FBS‐free DMEM at 37°C with 10% CO_2_. Cells were washed with Dulbecco's phosphate‐buffered saline (PBS) (Gibco, 14190144) and incubated in cDMEM for 5 days. Cell supernatants were collected, filtered, and aliquoted into sterile microtubes. Aliquots were stored at −80°C. Viral titers were determined using plaque assay.

### 4.3 CRISPR/Cas9 Gene‐Editing

Potential target sequences for CRISPR interference were identified using established design rules [51]. The sgRNAs were annealed and cloned into the chimeric CRISPR/Cas9 vector pX459 V2.0 (Addgene, #62988), which contains a puromycin resistant cassette. Sequences of generated pX459‐sgRNA clones were confirmed through Sanger sequencing. A549 and HEK293T cells were transfected with the pX459/UCHL3 sgRNA plasmid using TransIT‐LT1 (Mirus Bio, MIR2300) and subjected to puromycin selection (Gibco, A1113803) at a concentration of 4.0 µg/ml for 4 days. Single clones were isolated by limiting dilution, expanded, and validated for gene deletion by immunoblotting with anti‐UCHL3 antibody (Proteintech, 12384‐1‐AP) and by Sanger sequencing of the targeted locus. Two independent UCHL3^−/−^ clonal lines were used for all subsequent experiments to control for clonal artefacts.

### 4.4 Virus Infection

Cells were infected with ZIKV strain MR‐766/NC‐2014‐5132 or DENV2 (16681) at 1 multiplicity of infection (MOI), unless stated otherwise, and incubated for 1h in FBS‐free DMEM at 37°C with 10% CO_2_. Cells were washed with PBS and incubated in cDMEM at 37°C with 10% CO_2_. Cell supernatants were harvested at the specified time points, and viral titers were determined using plaque assay.

### 4.5 Plaque Assay

Harvested supernatants were 10‐fold serially diluted in FBS‐free DMEM and incubated with 5×10^5^/ml Vero cells (≥80% confluency) in 24‐well plates for 2 h at 37°C with 10% CO_2_. An overlay of 3% carboxymethyl cellulose (CMC) (Sigma) cDMEM was then added. After 84 h of incubation, the CMC overlay was aspirated, and cells were washed with PBS. Cells were fixed and stained with Amido black (1g Naphthol blue‐black powder, 60 ml glacial acetic acid, 13.6 g anhydrous sodium acetate in 1 l demineralized water) (Sigma) for 1 h, for quantification of plaque‐forming units (PFU). Samples were measured in technical triplicates.

### 4.6 Activity Based Protein Profiling

The HA‐Ub‐VME screen was performed as described previously [17]. ∼1×10^7^ cells were detached from 10 cm dishes by brief trypsinization, washed once with Hank's balanced salt solution (HBSS; Gibco, 14175095) and resuspended in 100 µl HBSS on ice. Perfringolysin O (PFO; a cholesterol‐dependent cytolysin used for selective plasma membrane permeabilization) [52, 53] was added to cells to a final concentration of 100 nM and maintained on ice for 5 min. The reaction mix was supplemented with an ATP regenerating mix, 10 µM HA‐Ub‐VME and protease inhibitor cocktail (Roche, 04693159001), and transferred to 37°C for 20 min. The reaction was terminated with lysis buffer. HA‐Ub‐VME reactive DUBs were resolved by SDS‐PAGE and visualized by silver staining.

### 4.7 Generation of UCHL3‐Reconstituted and Catalytic‐Dead Cell Lines

To generate stable UCHL3‐reconstituted cell lines, the Flag‐HA‐UCHL3 coding sequence was subcloned from the Flag‐HA‐UCHL3 plasmid (Addgene #22564) into the pLenti‐CMV‐Puro lentiviral expression vector. A catalytically inactive mutant was generated by site‐directed mutagenesis of the active‐site cysteine to alanine (C95A) using the Q5 Site‐Directed Mutagenesis Kit (NEB, E0554S), and the mutation was confirmed by Sanger sequencing. Lentiviral particles were produced by co‐transfecting HEK293T cells with the transfer plasmid, psPAX2 packaging plasmid (Addgene #12260), and pMD2.G envelope plasmid using TransIT‐LT1 reagent (Mirus Bio). Viral supernatants were collected at 48 and 72 h post‐transfection, filtered through 0.45 µm membranes, and used to transduce UCHL3^–/–^ A549 and HEK293T cells in the presence of 8 µg/ml polybrene (Sigma‐Aldrich, TR‐1003‐G). Transduced cells were selected with 4.0 µg/ml puromycin for 7 days, and stable expression of wild‐type or C95A Flag‐HA‐UCHL3 was confirmed by immunoblotting with anti‐Flag (Sigma‐Aldrich, F1804) and anti‐UCHL3 antibodies. Expression levels were compared to endogenous UCHL3 in parental wild‐type A549 cells to ensure physiologically relevant reconstitution. Two independent UCHL3^–/–^ clonal lines were transduced in parallel to confirm rescue across distinct clonal backgrounds.

### 4.8 Plasmid Transfection

The Flag‐HA‐UCHL3 (#22564), BASU (#107250), and RNA motif plasmid cloning backbone (#107253) plasmids were obtained from Addgene. To produce the ZIKV sfRNA plasmid, sfRNA from ZIKV strain MR‐766 was amplified using primers BSMBI sfRNA ZIKV (Table 1). The resulting amplicon and a control RNA motif plasmid cloning backbone were digested with the BsmBI‐v2 restriction enzyme (NEB) for 1 h at 55 °C. The digested fragments were purified and ligated using a DNA ligation kit (TaKaRa) at 4°C overnight. The recombinant ZIKV sfRNA plasmid was confirmed using DNA sequencing.

**TABLE 1:** Sequences.

Target	Forward	Reverse
Actin	TGGACTTCGAGCAAGAGATG	GAAGGAAGGCTGGAAGAGTG
ZIKV NS5	AGCTATGGGTGGAACATAGTTCG	CTTCAGGACTAGATGATGACTCACC
DENV2 NS5	ACAAGTCGAACAACCTGGTCCAT	GCCGCACCATTGGTCTTCTC
ZIKV sfRNA	AAACCAAGCTCATAGTCAGG	CACAGCTAGTCTCCAGTTCA
ISG15	ACAGCCATGGGCTGGGA	CCTTCAGCTCTGACACCGAC
CXCL10	GGTGAGAAGAGATGTCTGAATCC	GTCCATCCTTGGAAGCACTGCA
IRF7	TGGTCCTGGTGAAGCTGGAA	GATGTCGTCATAGAGGCTGTTGG
BSMBI sfRNA ZIKV	CAAGCTTGGAGACGGCACCAATCTTAGTGTTG	AAGAGCTAGAGACGAGAAACCATGGATTTCC
UCHL3 (sgRNA)	5′‐CACCGCCGCTGGAGGCCAATCCCG‐3′	5′‐AAACCGGGATTGGCCTCCAGCGGC‐3′

HEK293 cells were seeded at 2×10^5^ cells/well (≥80% confluency) in 12‐well plates in cDMEM and incubated for 24 h at 37°C with 10% CO_2_. 2.5 µg plasmid DNA was added to 250 µL opti‐MEM I Reduced‐Serum Medium (Mirus). The diluted DNA mixture was added to *Trans*IT‐LT1 reagent (Mirus) in a 1:2 ratio. The *Trans*IT‐LT1 reagent:DNA complexes were incubated for 15 min at room temperature, then distributed dropwise to the seeded cells. 24h after transfection, cells were infected with mock (FBS‐free DMEM) or ZIKV (MOI = 0.5) for 24 h or 48 h; at these time points, cells were harvested, washed with PBS, and analysed.

To establish dose‐dependence between UCHL3 expression levels and sfRNA accumulation, HEK293 cells were seeded at 2 × 10^5^ cells/well in 12‐well plates and incubated in cDMEM for 24 h at 37°C with 10% CO_2_. Cells were transfected with increasing amounts of Flag‐HA‐UCHL3 plasmid (0.25, 0.5, 1.0, 2.0, and 2.5 µg per well), with total DNA normalized to 2.5 µg per well using pcDNA3.1 empty vector. Transfection was performed using TransIT‐LT1 (Mirus Bio, MIR2300) as described above. 24 h after transfection, cells were infected with ZIKV (MR‐766, MOI 0.5). At 24 and 48 h post‐infection, cells were harvested for analysis. UCHL3 protein levels were assessed by immunoblotting with anti‐UCHL3 (Proteintech, 12384‐1‐AP, 1:1000) and anti‐Flag (Sigma‐Aldrich, F1804, 1:1000) antibodies, and sfRNA and NS5 genomic RNA levels were quantified by RT‐qPCR.

### 4.9 Interferon Treatment

A549 and A549 UCHL3^−/−^ cells were seeded at 2×10^5^ cells/well (≥80% confluency) in 12‐well plates and incubated in cDMEM for 24h at 37°C with 10% CO_2_. Cells were infected with mock (FBS‐free DMEM) or ZIKV (MOI = 0.5). 6 h post‐infection, cells were treated with 1000U/ml universal type‐I IFN alpha (PBL Assay Science #11200‐1) in cDMEM, for 24 h and 48 h; at these time points, cells were harvested, washed with PBS, and analyzed.

### 4.10 RT‐qPCR

Harvested cells were lysed with RLT buffer (QIAGEN) for RNA analysis. Total RNA was extracted using the RNeasy Mini Kit (QIAGEN). RNA concentrations were quantified using Nanodrop DS‐11 FX (DeNovix). RNA samples were diluted in demineralized water to yield a final concentration of 50 ng/µl. RNA samples were prepared using the One‐Step TB Green PrimeScript RT‐qPCR Kit (TaKaRa Bio). The Master Mix was made following the manufacturer's protocol, comprising 5 µl RT‐PCR Buffer III, 0.4 µl TaKaRa Ex Taq HS, 0.4 µl PrimeScript RT Enzyme Mix II, 2.2 µl RNase Free dH2O, and ROX Reference Dye 50×0.2 µl. The specified primers (Table 1) (Invitrogen) were diluted in the Master Mix. 9 µl of Master Mix and 1.5 µl of RNA sample were added per well in a MicroAmp Fast Optical 96‐Well Reaction Plate (Thermo Scientific). RNA transcripts were quantified using the ΔCT method, with actin as a reference, using the Applied Biosystems QuantStudio 3 Real‐Time PCR System (Thermo Scientific). Samples were measured in technical triplicates.

### 4.11 Immunofluorescence Assay

A549 and A549 UCHL3^−/−^ cells were seeded at 5×10^4^ cells/well (≥80% confluency) on 24‐well glass coverslips and incubated in cDMEM for 24 h at 37 °C with 10% CO_2_. Cells were infected with mock (FBS‐free DMEM) or ZIKV (MOI = 1) for 24 h. cDMEM was aspirated, and cells were washed with PBS. Cells were fixed with 4% paraformaldehyde (PFA) in PBS for 15 min at room temperature. PFA was aspirated, and cells were washed 3 times with PBS. Cells were permeabilized with 0.1% Triton X‐100 (Sigma) in PBS for 10 min at room temperature. 0.1% Triton X‐100/PBS was aspirated, and cells were washed with PBS. Cells were blocked with 2% BSA/PBS for 30 min at room temperature. Cells were probed with the specified primary antibodies diluted at 1:100 in 2% BSA/PBS, incubated overnight at 4°C. Cells were washed with PBS 3 times, each for 5 min. Cells were incubated in the dark with Alexa Fluor 555 goat anti‐mouse or 488 goat anti‐rabbit secondary antibodies (Table 2) for 1h at room temperature, diluted at 1:500 in 2% BSA/PBS. Cells were washed with PBS 3 times, each for 5 min. Coverslips were mounted onto glass slides using 6µL of Duolink in situ mounting medium with DAPI (Sigma) and exposed using a ZEISS LSM 880 confocal with Airyscan microscope (ZEISS). Images were analysed using ZEISS ZEN Microscopy Software (ZEISS), and fluorescent signal intensities were quantified using Image J.

**TABLE 2:** Antibodies.

Target	Host/Type	Manufacturer	Catalogue No.	Application
UCHL3	Rabbit polyclonal	Proteintech	12384‐1‐AP	WB 1:1000; IF 1:100; PLA 1:100
UCHL3 (mouse)	Mouse monoclonal	Santa Cruz	sc‐271002	WB 1:500
Flag (M2)	Mouse monoclonal	Sigma‐Aldrich	F1804	WB 1:1000; IP
HA	Rabbit polyclonal	Abcam	ab9110	WB 1:2000
NS5 (ZIKV)	Rabbit polyclonal	GeneTex	GTX133312	WB 1:1000
NS3 (ZIKV)	Rabbit polyclonal	GeneTex	GTX133309	WB 1:1000
Envelope (E)	Mouse monoclonal (4G2)	Millipore	MAB10216	WB 1:1000; IF 1:200
Capsid (C)	Rabbit polyclonal	GeneTex	GTX133317	WB 1:1000
prM	Rabbit polyclonal	GeneTex	GTX133305	WB 1:1000
NS4A	Rabbit polyclonal	GeneTex	GTX133704	WB 1:1000
dsRNA (J2)	Mouse monoclonal	Jena Bioscience / SCICONS	RNT‐SCI‐10010200	IF 1:200
G3BP1	Rabbit polyclonal	Proteintech	13057‐2‐AP	WB 1:1000
DCP1b	Mouse monoclonal	Santa Cruz	sc‐100706	IF 1:200; PLA 1:100
PABPC1	Rabbit polyclonal	Abcam	Ab21060	IF 1:200
PABPC1	Mouse monoclonal	Santa Cruz	sc‐32318	IF 1:100; PLA 1:100
RNase L	Mouse monoclonal	Santa Cruz	sc‐74405	WB 1:500
α‐Tubulin	Mouse monoclonal	Sigma‐Aldrich	T5168	WB 1:5000
β‐Tubulin	Mouse monoclonal	Sigma‐Aldrich	T8328	WB 1:5000
Vinculin	Mouse monoclonal	Sigma‐Aldrich	V9131	WB 1:5000
GAPDH	Mouse monoclonal	Santa Cruz	sc‐47724	WB 1:2000
Lamin B1	Rabbit polyclonal	Abcam	ab16048	WB 1:2000
Histone H3	Rabbit polyclonal	Abcam	ab1791	WB 1:5000
Alexa Fluor 488 goat anti‐rabbit	Goat polyclonal	Thermo Fisher	A‐11008	IF 1:500
Alexa Fluor 488 goat anti‐mouse	Goat polyclonal	Thermo Fisher	A‐11001	IF 1:500
Alexa Fluor 555 goat anti‐mouse	Goat polyclonal	Thermo Fisher	A‐21422	IF 1:500
Alexa Fluor 647 goat anti‐rabbit	Goat polyclonal	Thermo Fisher	A‐21244	IF 1:500
HRP goat anti‐rabbit	Goat polyclonal	Jackson ImmunoResearch	111‐035‐003	WB 1:10000
HRP goat anti‐mouse	Goat polyclonal	Jackson ImmunoResearch	115‐035‐003	WB 1:10000

### 4.12 Proximity Ligation Assay

Proximity ligation assays (PLA) were performed using the Duolink In Situ PLA kit (Sigma‐Aldrich, DUO92101) according to the manufacturer's protocol. A549 wild‐type cells were seeded on glass coverslips at 5×10^4^ cells/well and infected with ZIKV (MOI = 1) or mock‐infected for 24 h. Cells were fixed with 4% PFA for 15 min and permeabilized with 0.1% Triton X‐100 for 10 min. After blocking with Duolink blocking solution for 60 min at 37°C, cells were incubated overnight at 4°C with primary antibody pairs: rabbit anti‐UCHL3 (Proteintech, 12384‐1‐AP, 1:100) with mouse anti‐DCP1b (Santa Cruz, sc‐100706, 1:100), or rabbit anti‐UCHL3 with mouse anti‐PABPC1 (Santa Cruz, sc‐32318, 1:100). Duolink PLA PLUS and MINUS probes were applied for 60 min at 37°C, followed by ligation (30 min, 37°C) and amplification (100 min, 37°C) using the Duolink detection reagent (Far Red). Coverslips were mounted with Duolink in situ mounting medium with DAPI. Z‐stacks were acquired on a ZEISS LSM 880 confocal microscope with a 63× objective. PLA punctae were quantified using ImageJ across ≥50 cells per condition from 3 independent experiments.

### 4.13 ISRE‐Luciferase Reporter Assay

To assess the effect of UCHL3 on sfRNA‐mediated interferon antagonism, an interferon‐stimulated response element (ISRE) luciferase reporter assay was employed. A549 wild‐type and UCHL3^−/−^ cells were seeded at 1 × 10^5^ cells/well in 24‐well plates and incubated in cDMEM for 24 h at 37°C with 10% CO_2_. Cells were co‐transfected with the pISRE‐Luc reporter plasmid and the Renilla luciferase control plasmid pRL‐TK (Promega, E2241; 25 ng/well) as an internal normalization control, together with either the ZIKV sfRNA‐BoxB construct or the control RNA motif backbone (250 ng/well), using TransIT‐LT1 (Mirus Bio, MIR2300) at a 1:2 DNA:reagent ratio. Twenty‐four hours after transfection, cells were treated with 1,000 U/ml universal type‐I IFN (PBL Assay Science, 11200–1) for 16 h. Cells were lysed and luciferase activity was measured using the Dual‐Luciferase Reporter Assay System (Promega, E1910). Firefly luciferase values were normalised to Renilla luciferase activity to control for transfection efficiency. Data are expressed as fold induction relative to unstimulated empty‐vector controls. Three independent biological replicates were performed, each in technical triplicate.

### 4.14 sfRNA Detection by RNA Fluorescence In Situ Hybridization

A549 wild‐type and UCHL3^−/−^ cells were seeded on glass coverslips at 5 × 10^4^ cells/well in 24‐well plates and infected with ZIKV (MOI 1) for 24 h unless otherwise stated. Cells were fixed with 4% PFA (Electron Microscopy Sciences, 15710) for 15 min at room temperature, permeabilized with 0.5% Triton X‐100 in PBS for 10 min, and pre‐hybridized in wash buffer (2× SSC, 10% formamide) for 5 min. Stellaris FISH probes (Biosearch Technologies) targeting ZIKV 3' UTR sfRNA sequences, labelled with Quasar 570, were hybridized at 125 nM final concentration overnight at 37°C in hybridization buffer (10% dextran sulfate, 10% formamide, 2× SSC, 0.2 mg/ml BSA, 1 mg/ml yeast tRNA, and RNase inhibitor). Post‐hybridization washes comprised 2 × 30 min in wash buffer at 37 °C, followed by DAPI counterstaining and a final wash in 2× SSC.

For combined RNA‐FISH and immunofluorescence, cells were post‐fixed with 4% PFA for 10 min after the final FISH wash, blocked with 2% BSA in PBS for 30 min, and incubated overnight at 4°C with primary antibodies. After three PBS washes (5 min each), cells were incubated with secondary antibodies for 1 h at room temperature in the dark. Coverslips were mounted with ProLong Gold Antifade Mountant with DAPI (Thermo Fisher, P36931). Z‐stacks (0.3 µm steps) were acquired on a ZEISS LSM 880 confocal microscope with Airyscan using a 63× oil objective, and maximum intensity projections were generated.

Three antibody configurations were used. For sfRNA–DCP1b colocalization, mouse anti‐DCP1b (Santa Cruz, sc‐100706, 1:200) was detected with Alexa Fluor 488 goat anti‐mouse (Thermo Fisher, A‐11001, 1:500). For sfRNA–PABPC1 colocalization, rabbit anti‐PABPC1 (Abcam, ab21060, 1:200) was detected with Alexa Fluor 647 goat anti‐rabbit (Thermo Fisher, A‐21244, 1:500). These two experiments were performed as separate double‐labelling reactions rather than a single triple label, to avoid spectral bleedthrough between closely spaced emission spectra and to preserve epitope integrity following the hybridization protocol.

Colocalization was quantified using Manders' overlap coefficient calculated with the JACoP plugin in ImageJ on ≥50 cells per condition (≥100 cells for the epistasis experiments) from 3 independent experiments. Colocalization analysis was restricted to cytoplasmic sfRNA puncta; the perinuclear signal corresponding to viral replication organelles was excluded by masking the juxtanuclear compartment defined by the concentration of sfRNA signal intensity.

### 4.15 Quantification of rRNA Cleavage

For electrophoretic analysis of rRNA integrity, total RNA was extracted as described above and 500 ng per sample was resolved on a 1.2% agarose gel containing 1× MOPS buffer and 2.2 m formaldehyde. RNA loading was verified by UV spectrophotometry (A260/A280 ratio) prior to gel electrophoresis to ensure equivalent loading across all conditions. Band intensities of intact 28S and 18S rRNA species and cleavage products were quantified using ImageJ gel analysis tools. The RNase L activation index was calculated as the ratio of summed cleavage product intensity to summed intact (28S + 18S) rRNA intensity, following the method of Rath et al. [29]. Quantification was performed on three independent biological replicates and statistical significance was assessed by unpaired Student's t‐test with Welch's correction.

### 4.16 Immunoprecipitation Assay

HEK293 cells were seeded at 8×10^5^ cells/well (≥80% confluency) in 60 mm plates and incubated in cDMEM for 24 h at 37°C with 10% CO_2_. Cells were transfected with Flag‐HA‐UCHL3 plasmid or pcDNA3.1 (empty)‐TAG control plasmid for 24 h. Cells were infected with mock (FBS‐free DMEM) or ZIKV (MOI = 1) and incubated for 48 h. Cells were lysed using Lysis Buffer (50 mM HEPES pH 7.4, 150 mM NaCl, 1 mM MgCl2, 1% Triton X‐100). Cell lysates were cleared by centrifugation at 13,000 rpm for 10 min at 4°C. Cleared lysates were incubated with Anti‐FLAG M2 Magnetic Beads (Sigma), with rotation for 3 h at 4°C. The beads were washed 4 times in Lysis Buffer and resuspended in Sample Buffer. The resuspended beads were boiled for 10 min at 90°C. FLAG‐tagged UCHL3 protein and its interactors were analyzed by Western Blot.

### 4.17 sfRNA‐Interactome Capture Assay

A modified version of RNA‐protein interaction assay was employed to identify potential sfRNA‐interacting proteins [28]. ZIKV sfRNA flanked by BoxB stem loops are specifically recognized by a λN peptide fused to the N‐terminus of the BASU biotin ligase. The BoxB stem loops recruit the BASU fusion protein to the RNA, enabling biotinylating of proteins that interact with the sfRNA. The biotinylated proteins are isolated using streptavidin‐conjugated beads [28].

293 cells were seeded at 8×10^5^ cells/well (≥80% confluency) in 60 mm plates and incubated in cDMEM for 24h at 37 °C with 10% CO_2_. Cells were transfected with ZIKV sfRNA plasmid or control RNA motif plasmid cloning backbone, with BASU, for 24 h. Cells were infected with mock (FBS‐free DMEM) or ZIKV (MOI = 1) and incubated for 24 h. Cells were incubated with 200 µM of biotin (Sigma) for 1 h. Cells were lysed using Lysis Buffer. Cell lysates were cleared by centrifugation at 13,000 rpm for 10 min at 4 °C. Cleared lysates were incubated with Pierce Streptavidin Magnetic Beads (Thermo Scientific), with rotation for 3 h at 4°C. The beads were washed 4 times in Lysis Buffer and resuspended in Sample Buffer. The resuspended beads were boiled for 10 min at 90°C. Biotinylated proteins were analyzed by Western Blot.

To identify proteins associated with sfRNA‐containing ribonucleoprotein complexes, quantitative proteomic analysis was performed on streptavidin‐affinity captured fractions from cells expressing either ZIKV sfRNA‐BoxB or control RNA motif constructs alongside BASU. For each condition, three biological replicates of streptavidin pulldown samples and corresponding control samples (cells expressing control RNA backbone with BASU) were analyzed. To minimize nonspecific binding, protein abundance in sfRNA‐expressing samples was compared with that in control RNA samples.

Protein digestion, labelling, and peptide fractionation were conducted according to standard protocols at the Bristol Proteomics Facility (https://www.bristol.ac.uk/life‐sciences/research/facilities/proteomics/) using the TMTsixplex isobaric labelling kit (Thermo Fisher Scientific). Peptides were analyzed by LC–MS/MS on a Q Exactive Plus Hybrid Quadrupole‐Orbitrap mass spectrometer (Thermo Fisher Scientific). Raw data were processed using Proteome Discoverer v2.5 (Thermo Scientific) with the UniProt Human database, applying a false discovery rate (FDR) < 1% at both the peptide and protein levels. Protein quantification was normalized to total peptide abundance across all channels.

For statistical analysis, proteins with a log_2_ fold change (FC) ≥ 1 and P ≤ 0.05 (Student's t‐test) were considered significantly enriched relative to the control RNA condition.

### 4.18 RNase L Activation Assay

A549 wild‐type (WT), UCHL3^−/−^, UCHL3^WT^, and UCHL3^C95A^ cells were seeded 24 h before infection and inoculated with ZIKV (MR766 at MOI 2) for 1 h at 37 °C. At 24 h post‐infection, cells were washed twice with ice‐cold PBS and lysed on ice in 1× RNase‐free lysis buffer (50 mM Tris‐HCl, pH 7.5, 150 mM NaCl, 2 mM MgCl_2_, 1 mM DTT) supplemented with protease inhibitor cocktail and RNase inhibitor. Lysates were clarified (14,000xg, 10 min, 4°C), protein‐normalized (BCA), and maintained on ice. RNase L activity was quantified using a fluorogenic FRET RNA substrate in a 96‐well format as described before [54]. 10 µl of normalized lysate was combined with 50 nM recombinant human RNase L and 200 nM of a dual‐labelled RNA substrate (5’‐6‐FAM‐UUAUU‐BHQ‐1‐3’; Integrated DNA Technologies) in 50 µl total reaction volume of assay buffer (25 mM Tris‐HCl, pH 7.4, 100 mM KCl, 10 mM MgCl_2_, 1 mM DTT, 50 µM ATP). Endogenous 2–5A present in the cell lysate activates recombinant RNase L, which cleaves the dual‐labelled RNA reporter between the fluorophore (6‐FAM) and quencher (BHQ‐1), generating fluorescence proportional to 2–5A concentration. Fluorescence (excitation 485 nm, emission 520 nm) was measured kinetically at 37°C every 2 min for 60 min using plate reader. Initial cleavage rates (V0) were calculated from the linear portion of the fluorescence time course (typically 5–20 min). A standard curve was generated in parallel by adding serial dilutions (0.01–100 nM) of synthetic (2’‐5’)‐p3A3 (2‐5A trimer; Sigma‐Aldrich, A0788) to reaction mixtures containing recombinant RNase L and reporter substrate but using lysis buffer in place of cell lysate. Endogenous 2–5A concentrations were interpolated from the standard curve. Data were normalized to wild‐type mock‐infected controls and expressed as fold change in RNase L activity. All samples were measured in technical triplicates across three independent biological replicates.

### 4.19 Bioanalyzer Analysis of rRNA Integrity

To assess the temporal dynamics of RNase L activation, total RNA was extracted from A549 wild‐type and UCHL3^−/−^ cells at 6, 12, 18, and 24 h post‐infection with ZIKV (MR‐766, MOI 2) using the RNeasy Mini Kit (QIAGEN, 74104). RNA integrity was assessed using an Agilent 2100 Bioanalyzer with the RNA 6000 Nano Kit (Agilent Technologies, 5067‐1511) according to the manufacturer's protocol. One microlitre of total RNA (25–500 ng/µl) was loaded per well. Electropherograms were analyzed using Agilent 2100 Software. The RNase L activation index was calculated as the ratio of the integrated area of rRNA cleavage products (fragments migrating below the 18S peak) to the sum of intact 28S and 18S rRNA peak areas, following the quantification method described by Rath et al. [29]. Three independent biological replicates were analyzed per condition, and statistical comparisons were performed by two‐way ANOVA with Dunnett's post hoc test.

### 4.20 Statistical Analysis

Data are presented as the mean ± standard deviation (SD), unless stated otherwise. Statistical analyses were performed using GraphPad Prism 10. Significance was calculated using the unpaired Student's two‐tailed *t‐*test, with Welch's correction *t‐*test where applicable, or two‐way ANOVA with post‐hoc Dunnett's multiple comparisons test.

## Conflicts of Interest

The authors declare no conflicts of interest.

## Author Contributions

S.S. conceived and designed the study, O.T‐C., A.B‐D., Q.W.T, L.S, M.Y.L, performed the experiments and analyzed the data. K.J.H performed the proteomics experiments and A.P‐F analyzed the data. S.S and O.T‐C drafted the manuscript. All authors have reviewed and approved the final version of the manuscript.

## Supporting information




**Supporting File 1**: advs75949‐sup‐0001‐SuppMat.pdf.

**Supporting File 2**: advs75949‐sup‐0002‐blots.zip.

**Supporting File 3**: advs75949‐sup‐0003‐TableS1.docx.

**Supporting File 4**: advs75949‐sup‐0004‐FigureS1.tif.

**Supporting File 5**: advs75949‐sup‐0005‐FigureS2.tif.

**Supporting File 6**: advs75949‐sup‐0006‐FigureS3.tif.

**Supporting File 7**: advs75949‐sup‐0007‐FigureS4.tif.

## Data Availability

The complete mass spectrometry dataset for the sfRNA‐biotin interactome (Figure 3E) has been deposited in the PRIDE proteomics repository (https://www.ebi.ac.uk/pride/) under accession number PXD075983. Uncropped images of all Western blots are provided as supplementary material. All other data supporting the findings of this study are available from the corresponding author upon reasonable request.
