# Supplementary material for: UCHL3 Regulates Subgenomic Flaviviral RNA Condensates to Promote Virus Propagation
Source: Adv Sci (Weinh). 2026 Jun 3;13(48):e21781. doi: 10.1002/advs.202521781 (PMC13336449; doi:10.1002/advs.202521781)
Supplement: Supplementary file 1 [file ADVS-13-e21781-s004.pdf]

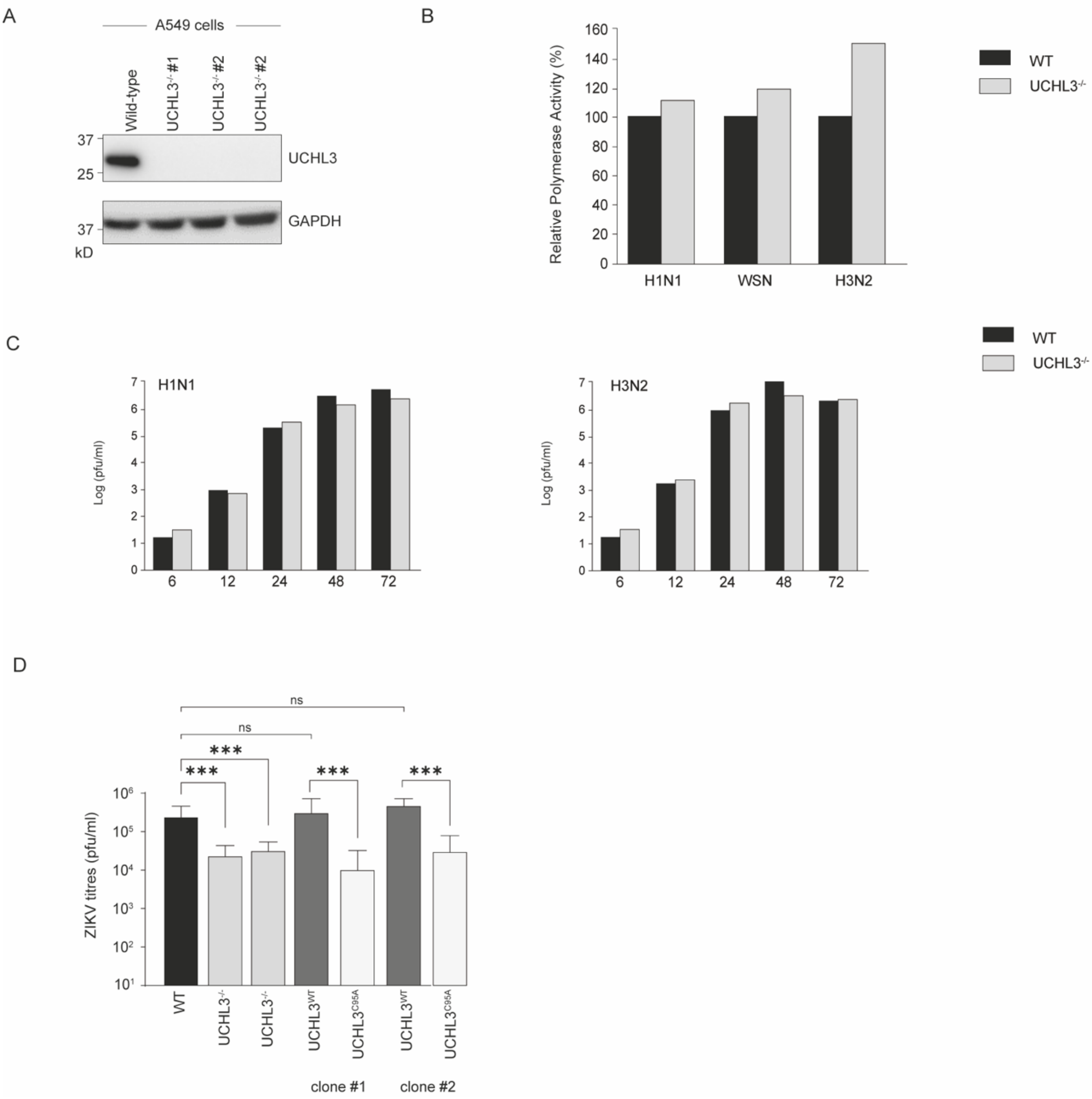

Supplementary Figure S1. UCHL3 deficiency exhibits virus-specific effects on replication.

(A) Immunoblot analysis of UCHL3 protein expression in two independent CRISPR-Cas9-generated A549 knockout clones (UCHL3$^{-/-}$ #1, #2) compared to parental wild-type cells. GAPDH serves as loading control.

(B) Minigenome-based analysis of influenza A virus polymerase activity in WT and UCHL3$^{-/-}$ A549 cells across three viral strains (H1N1, WSN, H3N2). Relative polymerase activity is expressed as a percentage of wild-type control levels.

(C) Multi-step growth curves comparing infectious virus production between WT and UCHL3$^{-/-}$ cells for H1N1 (left) and H3N2 (right) influenza strains. Viral titres (log pfu/ml) were determined at 6, 12, 24, 48, and 72 hours post-infection, revealing no substantial difference in replication kinetics.

(D) Multi-clone validation and catalytic activity-dependent rescue of the ZIKV replication phenotype. ZIKV titres (pfu/ml, log scale; New Caledonia strain, MOI 1) at 24 hours post-infection in WT, $UCHL3^{-/-}$, $UCHL3^{WT}$-reconstituted, and $UCHL3^{C95A}$ catalytic-mutant-expressing A549 cells from two independent knockout clones. Statistical significance determined by two-way ANOVA with Dunnett's multiple comparisons test; ***$p < 0.001$; ns, not significant.

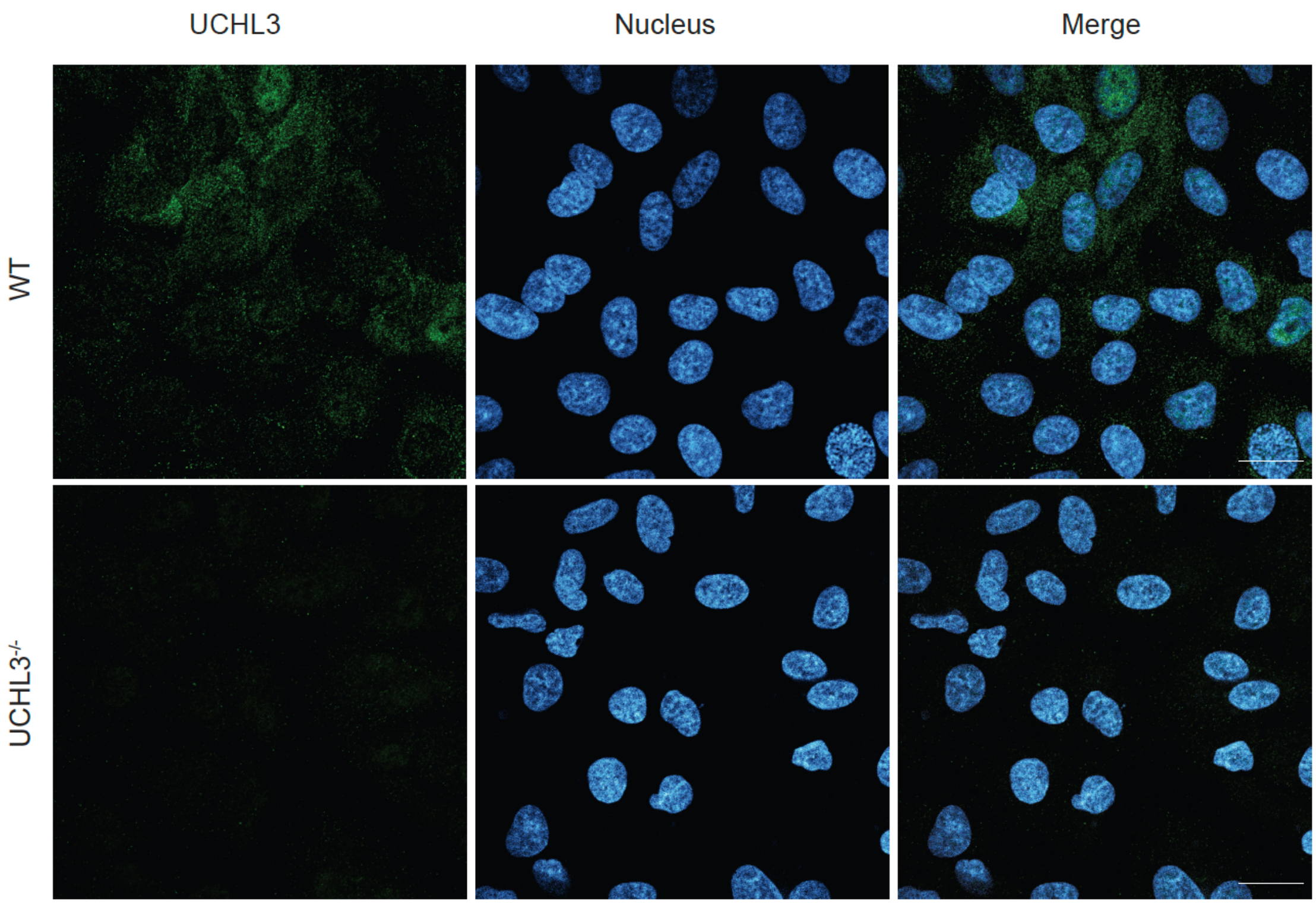

Supplementary Figure S2. Validation of UCHL3 antibody specificity in knockout cell lines.

Immunofluorescence analysis demonstrates antibody specificity and confirms complete UCHL3 ablation in CRISPR-Cas9-generated knockout cells. Representative confocal microscopy images of wild-type (WT, upper panel) and UCHL3$^{-/-}$ (lower panel) A549 cells stained for UCHL3 (green) with nuclei counterstained using DAPI (blue). Scale bar represents 20 μm.

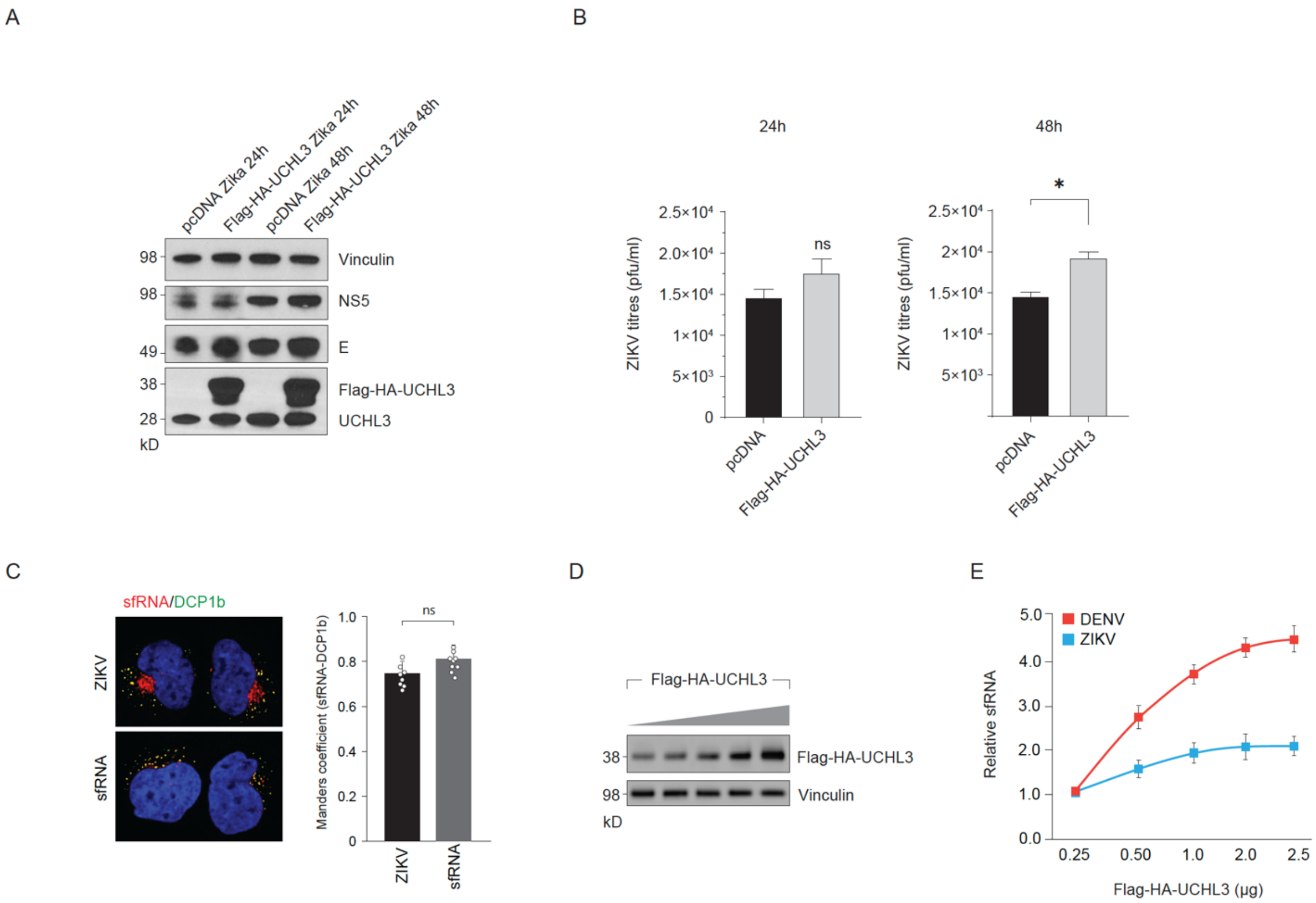

Supplementary Figure S3. Ectopic UCHL3 expression enhances flavivirus replication, viral protein accumulation, and sfRNA levels in a dose-dependent manner

(A) Immunoblot analysis of HEK293 cells transfected with pcDNA3.1 empty vector or Flag-HA-UCHL3 and subsequently infected with ZIKV (MOI 0.5). Lysates were collected at 24 and 48 hours post-infection and probed for viral proteins NS5 and envelope (E), Flag-HA-UCHL3, and endogenous UCHL3. Vinculin serves as a loading control.

(B) ZIKV titres (pfu/ml) determined by plaque assay in Flag-HA-UCHL3-transfected versus pcDNA3.1 control-transfected cells at 24 and 48 hours post-infection. Data represent mean ± SD from three independent experiments. *$p < 0.05$; ns, not significant.

(C) Fluorescence microscopy analysis of sfRNA (red) and DCP1b (green) co-localisation in ZIKV-infected cells compared with cells transfected with sfRNA alone. Nuclei are stained with DAPI (blue). Quantification of the Manders coefficient (sfRNA–DCP1b) shows no significant difference between ZIKV-infected and sfRNA-transfected conditions.

(D) Immunoblot confirming dose-dependent expression of Flag-HA-UCHL3 across an increasing transfection titration series. Vinculin serves as a loading control.

(E) Dose-response analysis of relative sfRNA levels as a function of increasing Flag-HA-UCHL3 expression (μg) for DENV (red) and ZIKV (blue). Both viruses show dose-dependent increases in sfRNA accumulation, with DENV exhibiting a greater magnitude of response. Data represent mean ± SEM.

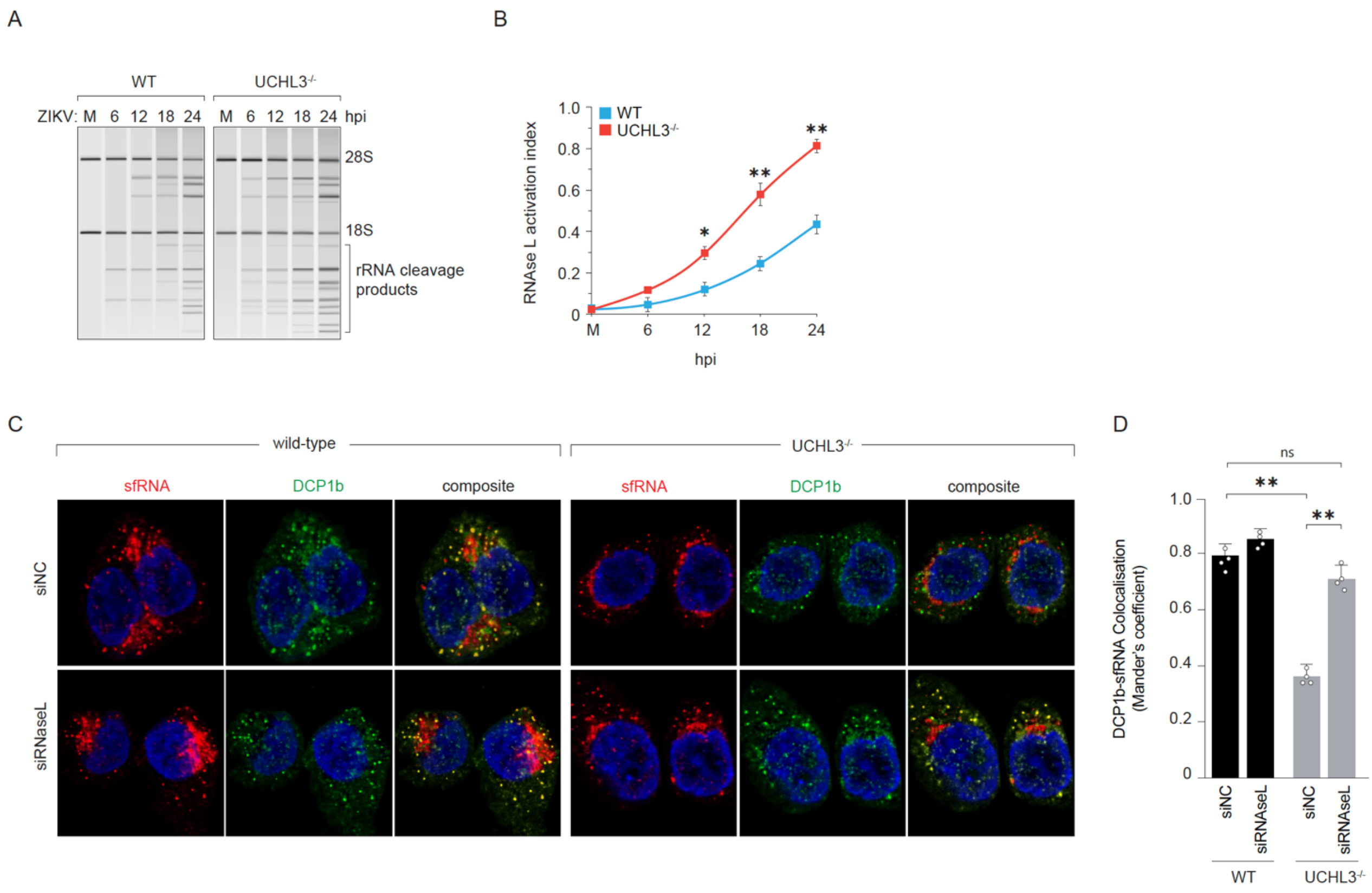

Supplementary Figure S4. UCHL3 deficiency accelerates RNase L activation during ZIKV infection and modulates sfRNA–DCP1b colocalisation.

(A) RNA integrity analysis of total RNA from WT and UCHL3-/- A549 cells infected with ZIKV across a time course (mock, 6, 12, 18, and 24 hours post-infection). Intact 28S and 18S ribosomal RNA bands and lower molecular weight rRNA cleavage products indicative of RNase L activation indicated.

(B) Quantification of the RNase L activation index over the ZIKV infection time course in WT (blue) and UCHL3-/- (red) cells. UCHL3-/- cells exhibit significantly elevated RNase L activation from 12 hpi onwards relative to WT controls. Statistical significance determined by two-way ANOVA with Dunnett's post hoc test. Data represent mean ± SEM. *$p < 0.05$; **$p < 0.01$.

(C) Representative fluorescence microscopy images of sfRNA (red), DCP1b (green), and composite merge (with DAPI, blue) in WT and UCHL3-/- cells treated with non-targeting control siRNA (siNC) or RNase L-targeting siRNA (siRNaseL).

(D) Quantification of DCP1b–sfRNA colocalisation by Manders coefficient. UCHL3-/- cells show markedly reduced sfRNA–DCP1b colocalisation compared to WT controls. RNase L knockdown has little effect on colocalisation in WT cells but significantly restores sfRNA–DCP1b colocalisation in UCHL3-/- cells. Data represent mean ± SEM. **$p < 0.01$; ns, not significant.
