# Supplementary material for: UCHL3 Regulates Subgenomic Flaviviral RNA Condensates to Promote Virus Propagation
Source: Adv Sci (Weinh). 2026 Jun 3:e21781. Online ahead of print. doi: 10.1002/advs.202521781 (PMC13336449; doi:10.1002/advs.202521781)
Supplement: Supplementary file 2 — Supporting File: advs75949‐sup‐0002‐blots.zip. [file ADVS-9999-e21781-s004.zip › advs75949-sup-0002-blots/Western blot source data.pdf]

Anti-UCHL3

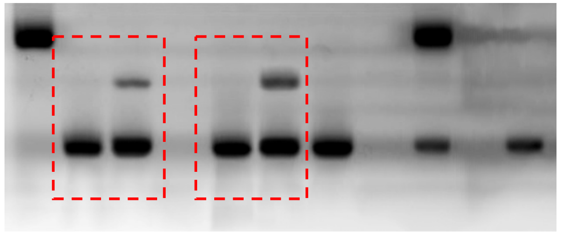

FIGURE 1A

Anti-Vinculin

Anti-E

Anti-UCHL3

Anti-Vinculin

Anti-UCHL3

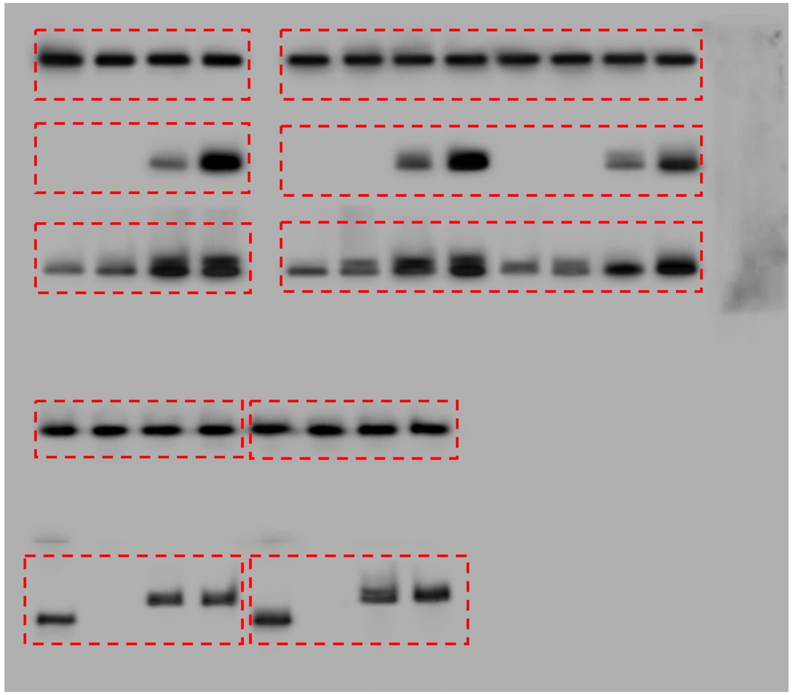

FIGURE 1B + 1F

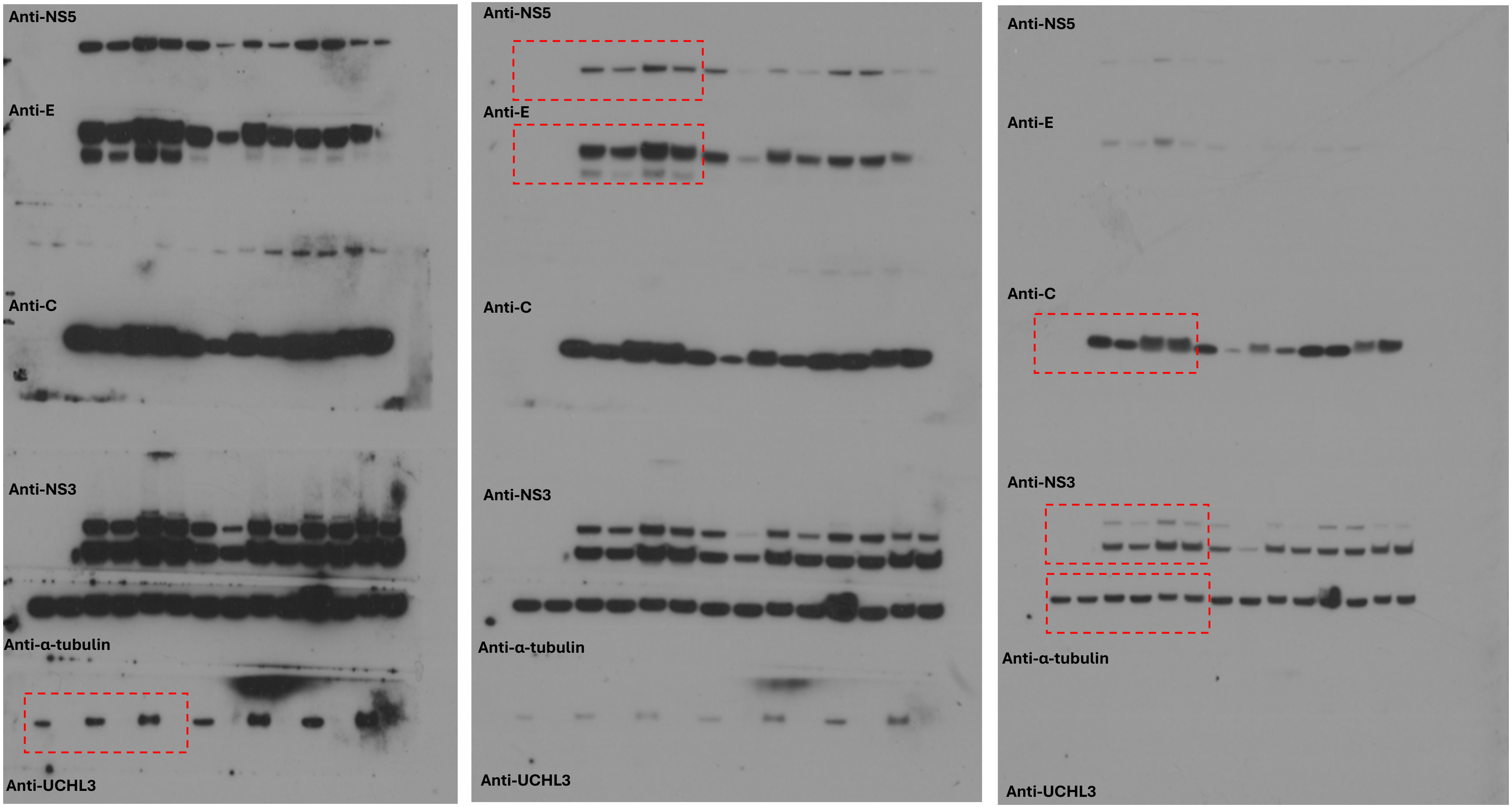
Anti-NS5
Anti-E
Anti-C
Anti-NS3
Anti-α-tubulin
Anti-UCHL3
Anti-NS5
Anti-E
Anti-C
Anti-NS3
Anti-α-tubulin
Anti-UCHL3
Anti-NS5
Anti-E
Anti-C
Anti-NS3
Anti-α-tubulin
Anti-UCHL3

FIGURE 1E

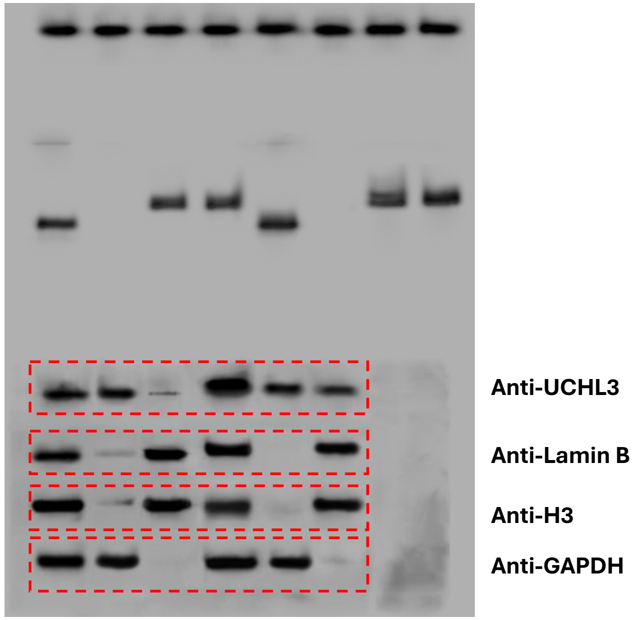

FIGURE 2C

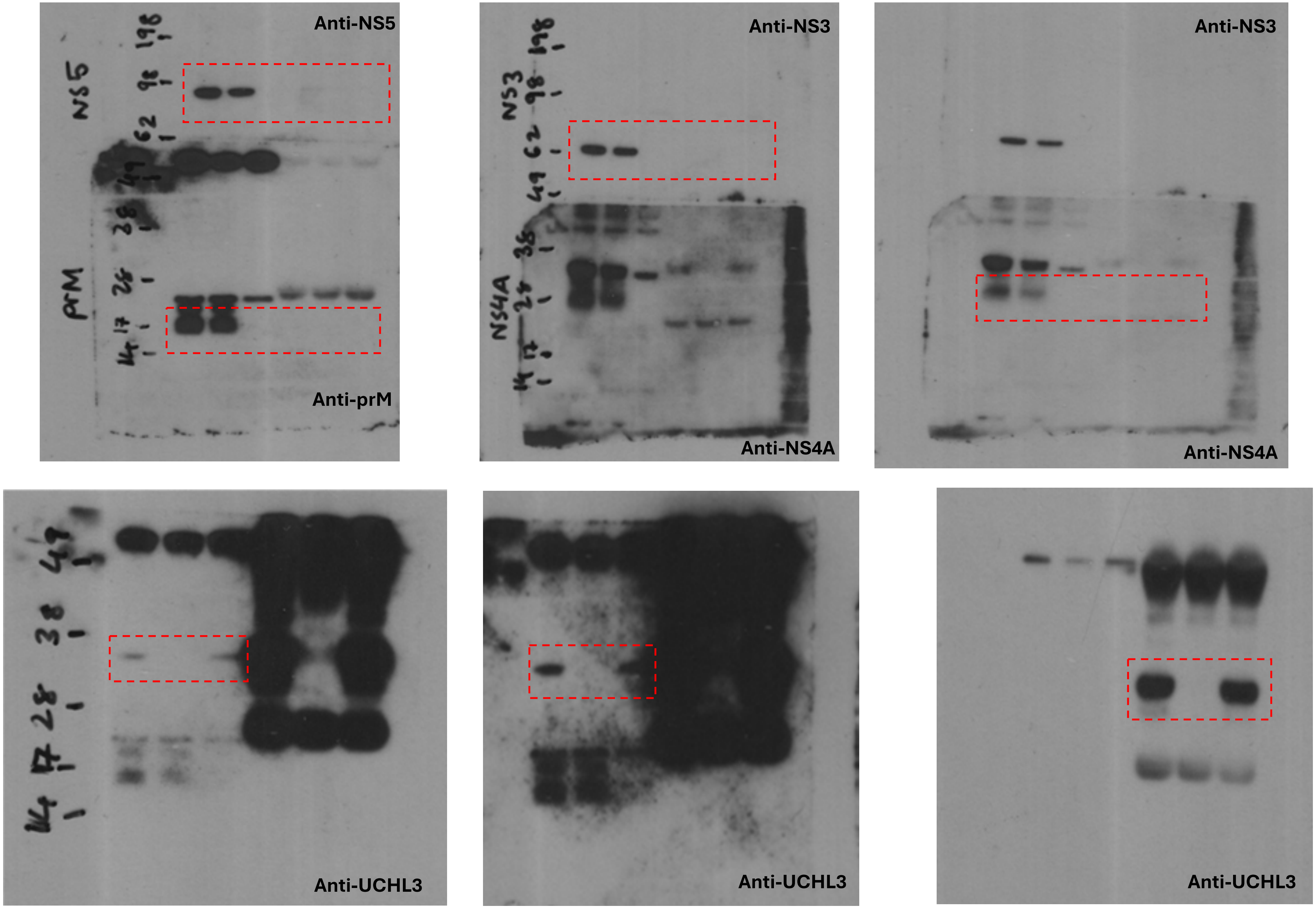
Anti-NS5
Anti-prM
Anti-NS3
Anti-NS4A
Anti-NS3
Anti-NS4A
Anti-UCHL3
Anti-UCHL3
Anti-UCHL3

FIGURE 3A

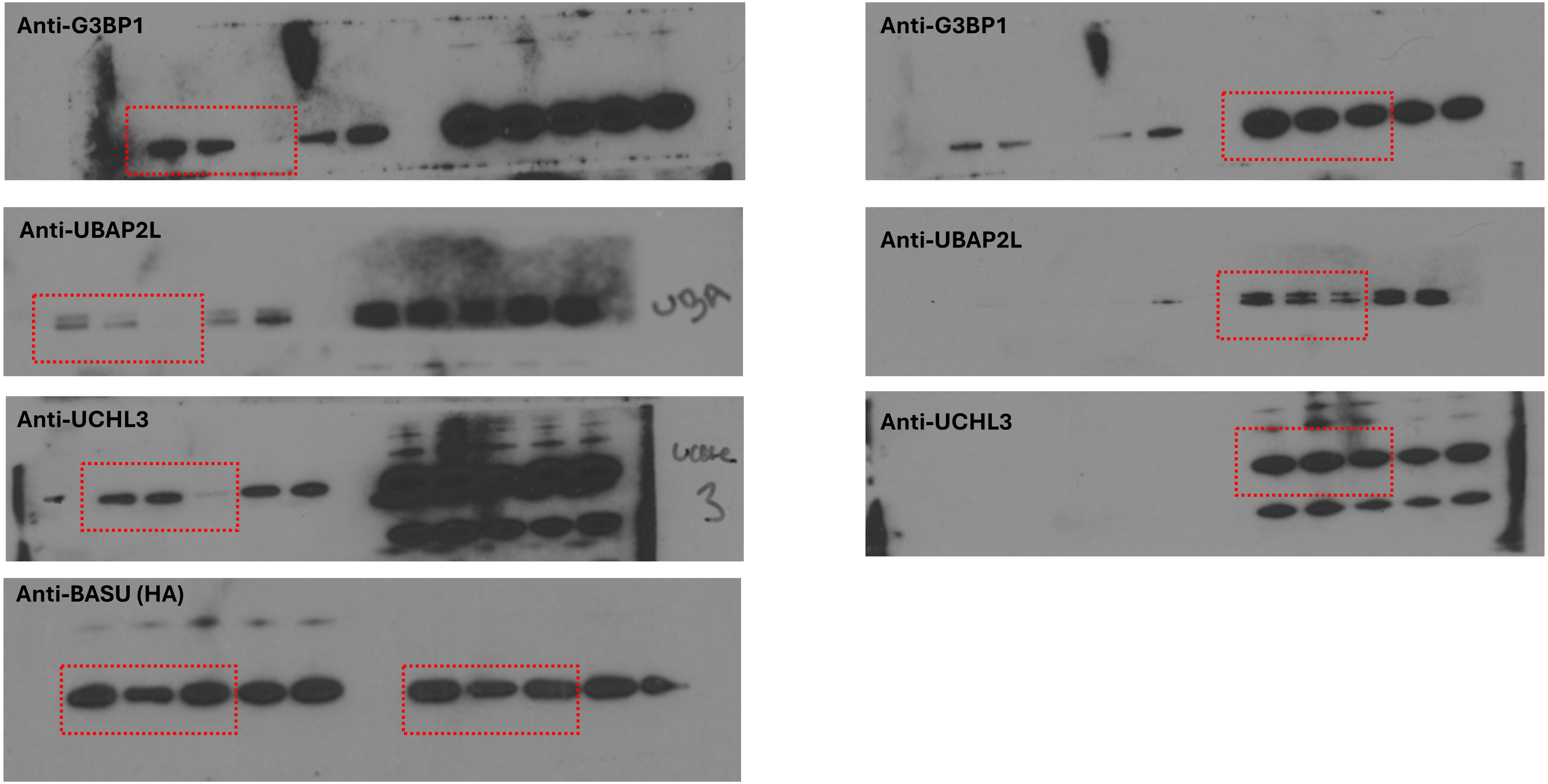
Anti-G3BP1
Anti-UBAP2L
Anti-UCHL3
Anti-BASU (HA)
Anti-G3BP1
Anti-UBAP2L
Anti-UCHL3

FIGURE 3D

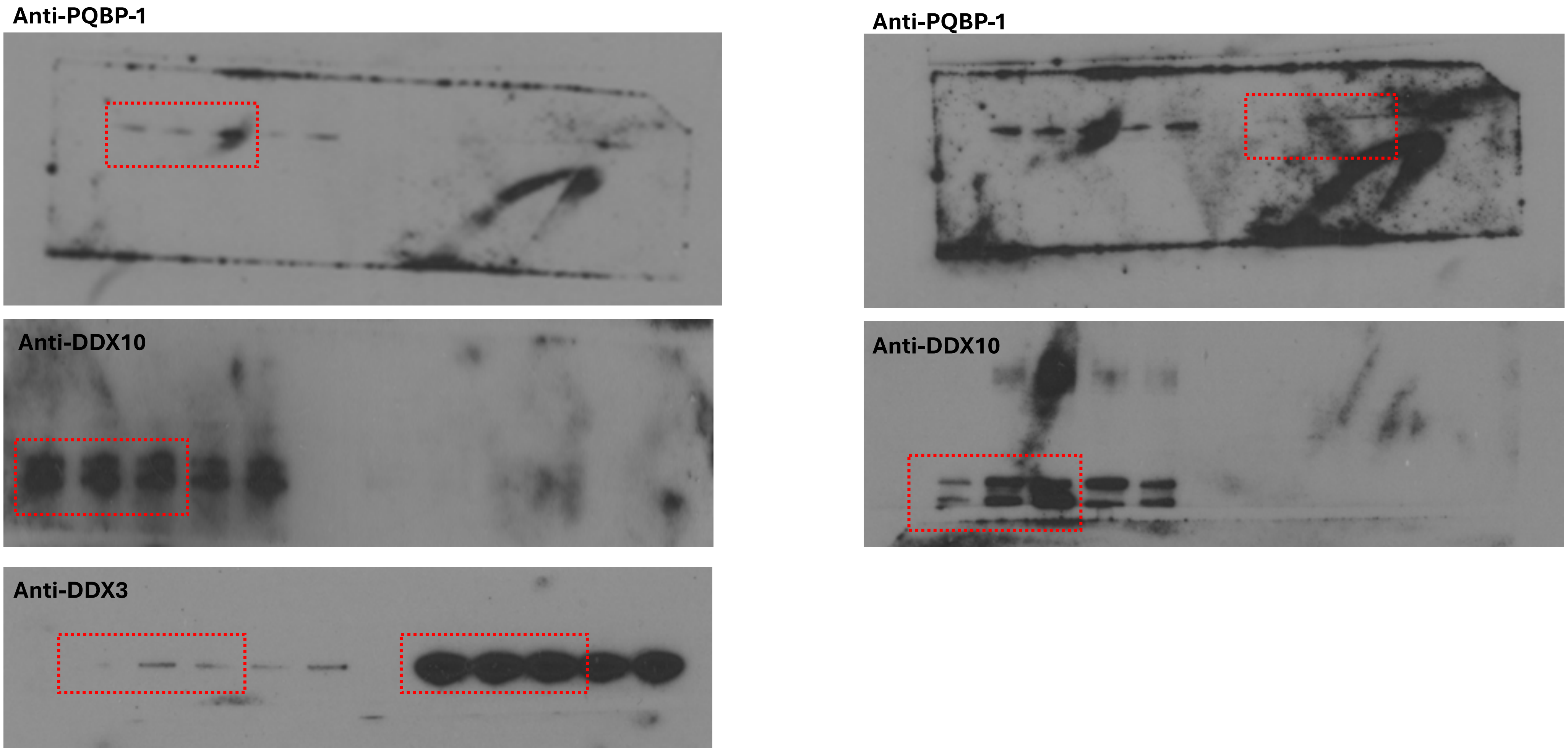

FIGURE 3D

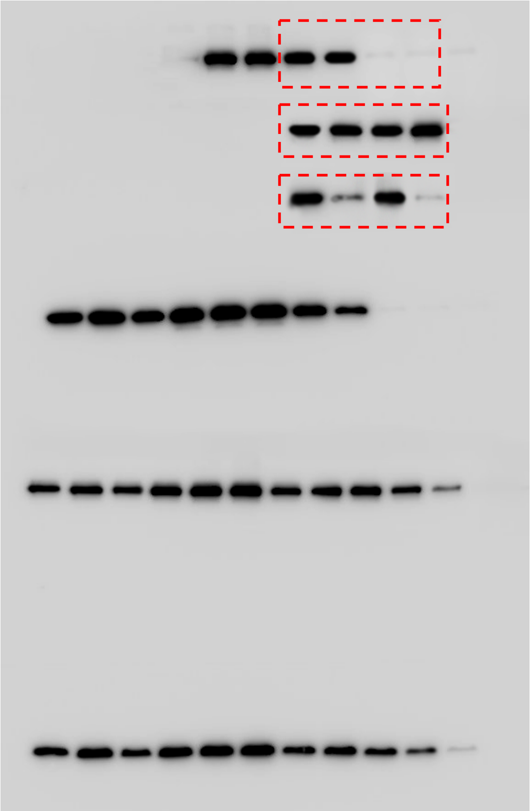

Anti-UCHL3

Anti-β-Tubulin

Anti-RNAseL

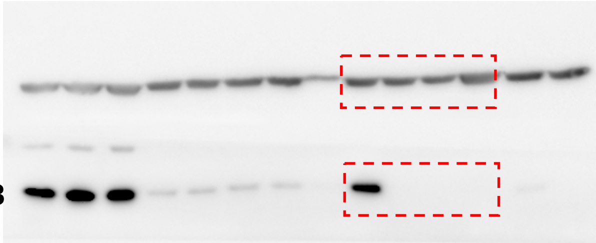

Anti-GAPDH

Anti-UCHL3

FIGURE S1A

FIGURE 4I

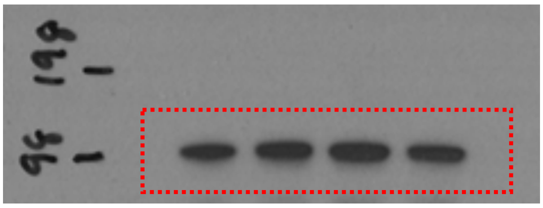

Anti-Vinculin

Anti-UCHL3

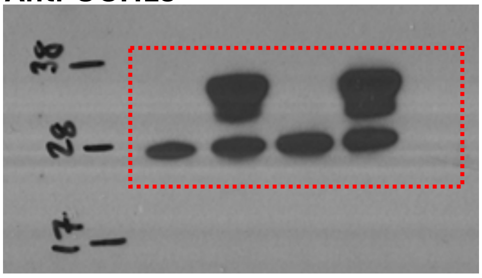

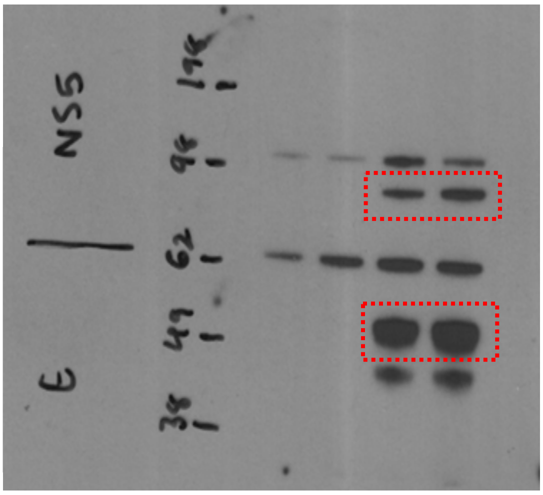

Anti-NS5

Anti-E

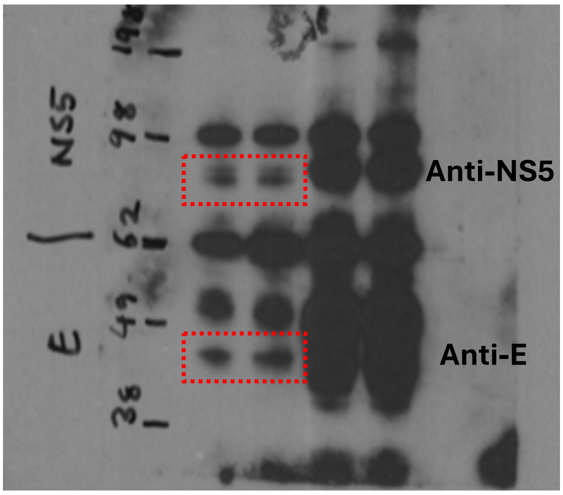

Anti-NS5

Anti-E

FIGURE S3A

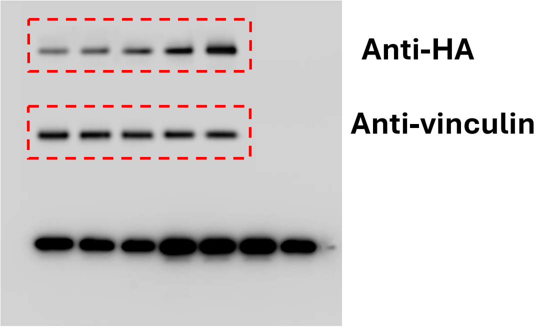

FIGURE S3D
