# Supplementary material for: UCHL3 Regulates Subgenomic Flaviviral RNA Condensates to Promote Virus Propagation
Source: Adv Sci (Weinh). 2026 Jun 3:e21781. Online ahead of print. doi: 10.1002/advs.202521781 (PMC13336449; doi:10.1002/advs.202521781)
Supplement: Supplementary file 3 — Supporting File: advs75949‐sup‐0003‐TableS1.docx. [file ADVS-9999-e21781-s002.docx]

**Table S1.** Identification of deubiquitylases by HA-Ub-VME activity-based profiling.

| **Accession** | **Protein** | **Family** | **Mass (kDa)** | **IAV** | **ZIKV** | **DENV** |
| --- | --- | --- | --- | --- | --- | --- |
| Q96FW1 | OTUB1 | OTU | 37 | + | + | + |
| Q9UHP3 | USP25 | USP | 122 | + | – | – |
| Q96RU2 | USP28 | USP | 122 | + | – | – |
| Q9Y614 | USP3 | USP | 59 | + | – | – |
| Q92560 | BAP1 | UCH | 80 | + | – | – |
| Q93009 | USP7 | USP | 128 | – | + | + |
| P54578 | USP14 | USP | 56 | – | + | + |
| Q9Y5K5 | UCHL5 | UCH | 38 | + | + | + |
| P15374 | UCHL3 | UCH | 26 | + | + | + |
| Q96G74 | OTUD5 | OTU | 61 | – | + | + |
| Q01804 | OTUD4 | OTU | 124 | – | + | + |
| Q7L8S5 | OTUD6A | OTU | 33 | + | – | + |
| Q9NQC7 | CYLD | USP | 107 | + | – | – |
| P21580 | TNFAIP3 (A20) | OTU | 90 | + | – | – |
| Q96DC9 | OTUB2 | OTU | 27 | + | – | – |
| Q9Y4E8 | USP15 | USP | 112 | + | – | – |
