# Supplementary figures and images for: UCHL3 Regulates Subgenomic Flaviviral RNA Condensates to Promote Virus Propagation
Source: Adv Sci (Weinh). 2026 Jun 3:e21781. Online ahead of print. doi: 10.1002/advs.202521781 (PMC13336449; doi:10.1002/advs.202521781)

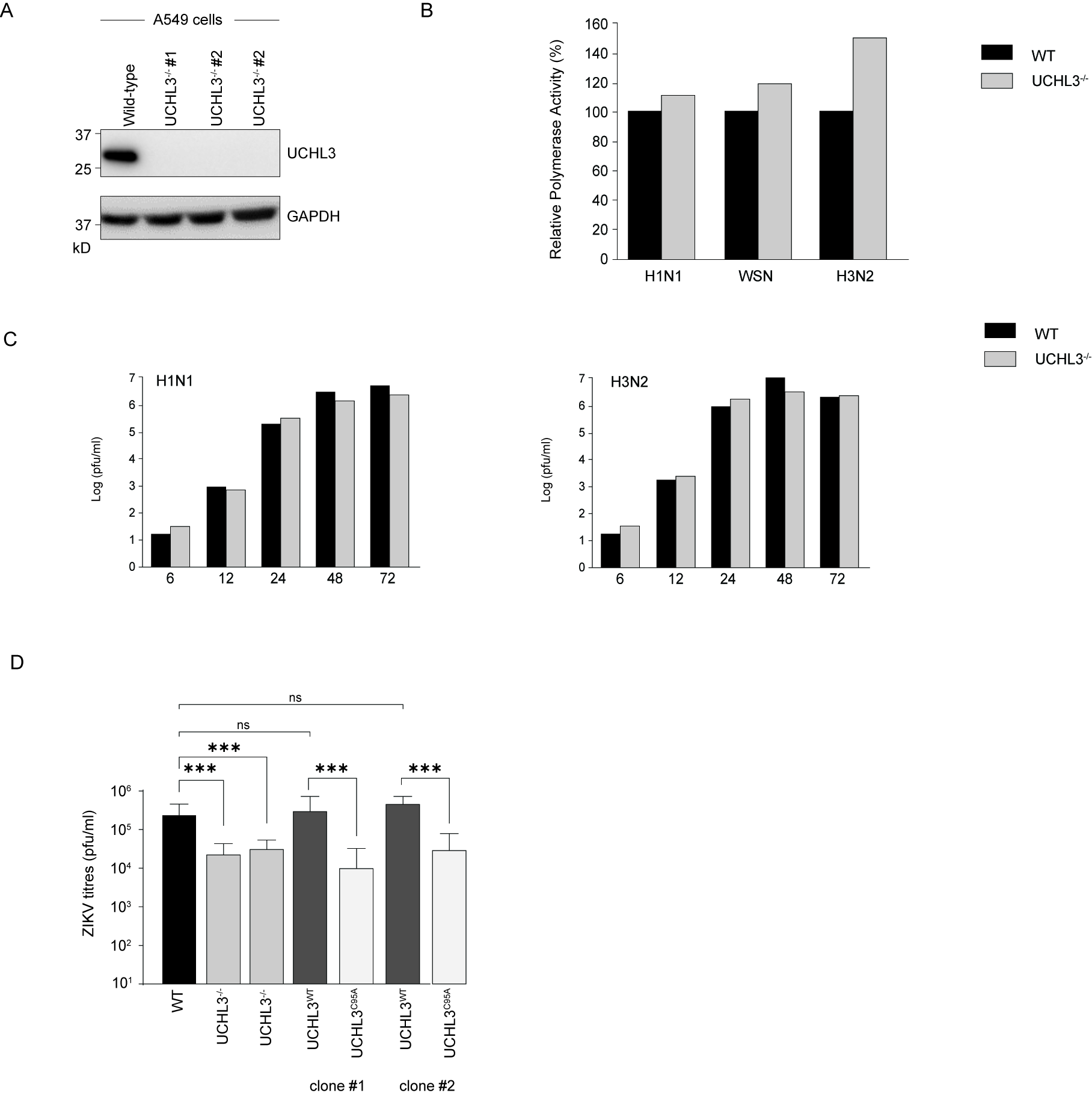

Supplement: Supplementary file 4 — Supporting File: advs75949‐sup‐0004‐FigureS1.tif. [file ADVS-9999-e21781-s003.tif]

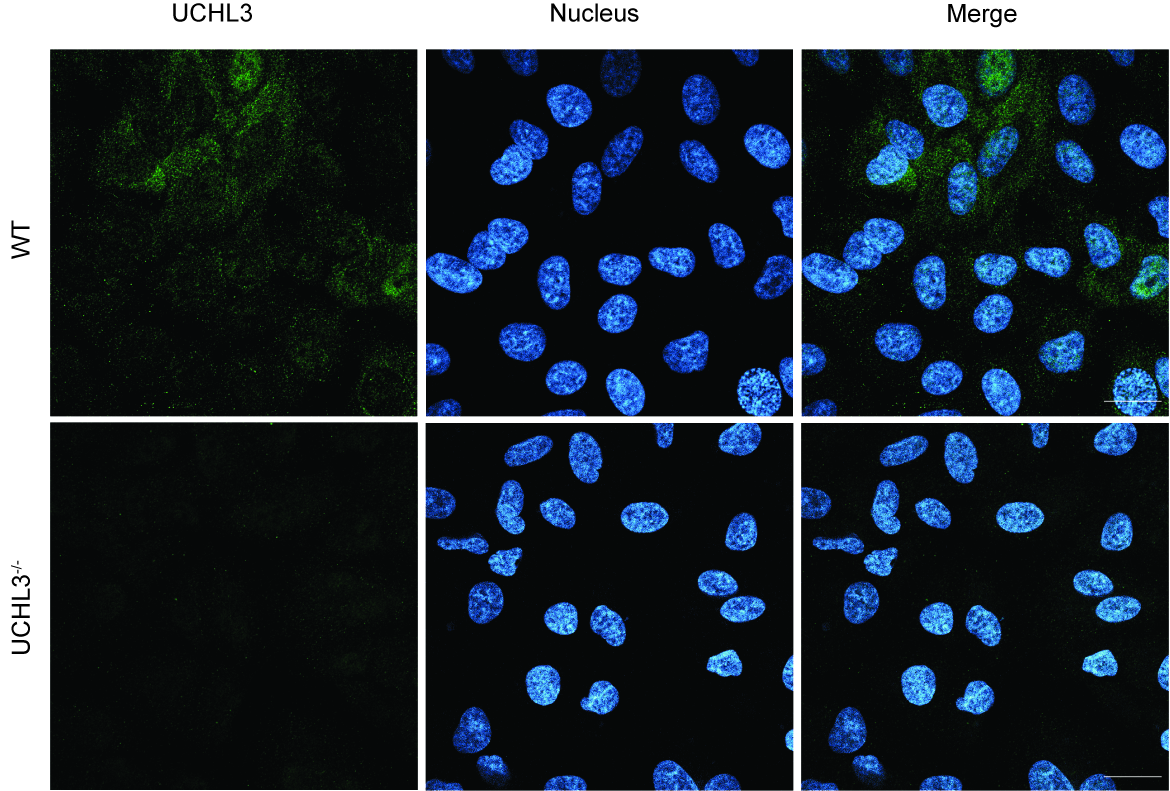

Supplement: Supplementary file 5 — Supporting File: advs75949‐sup‐0005‐FigureS2.tif. [file ADVS-9999-e21781-s001.tif]

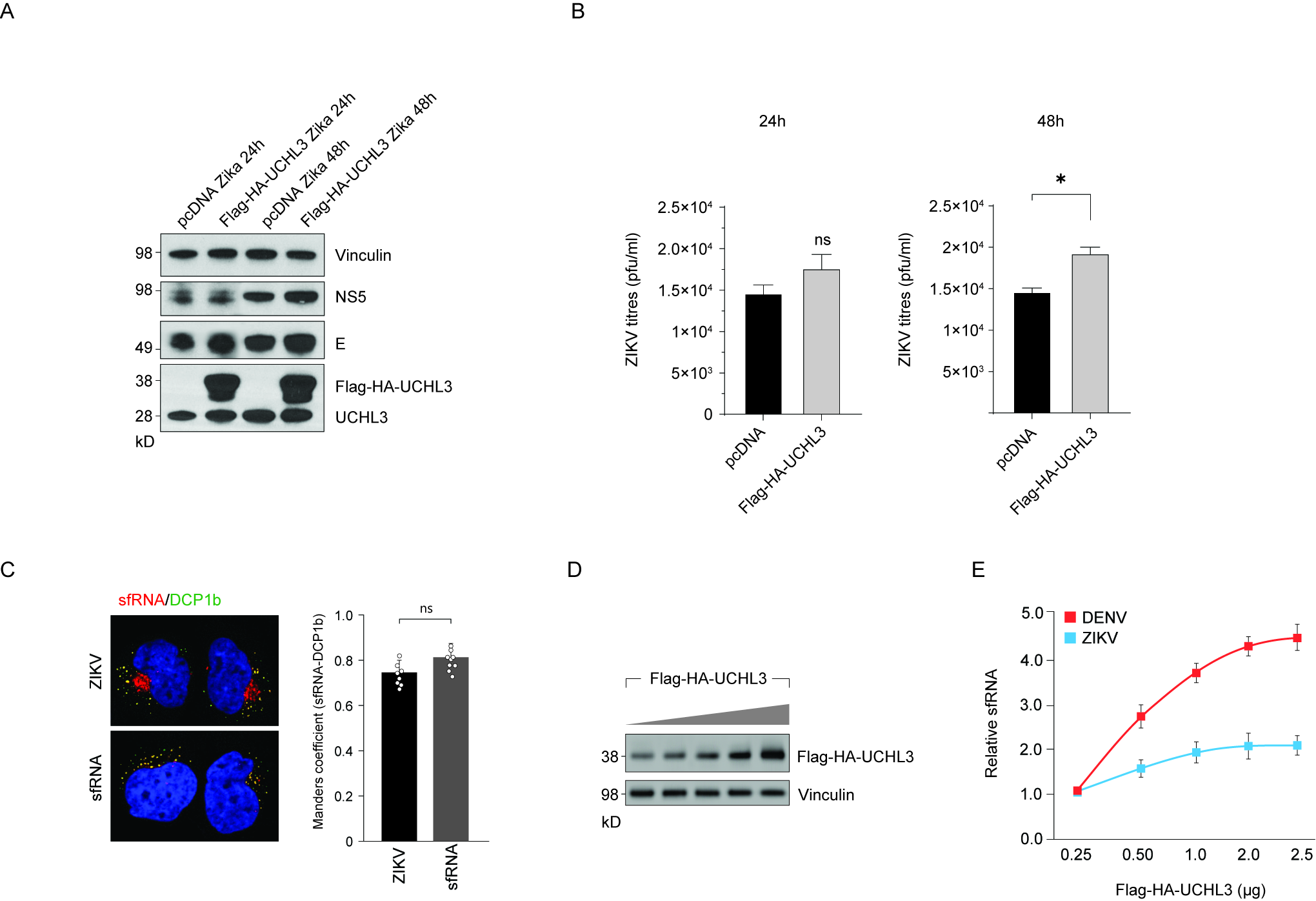

Supplement: Supplementary file 6 — Supporting File: advs75949‐sup‐0006‐FigureS3.tif. [file ADVS-9999-e21781-s007.tif]

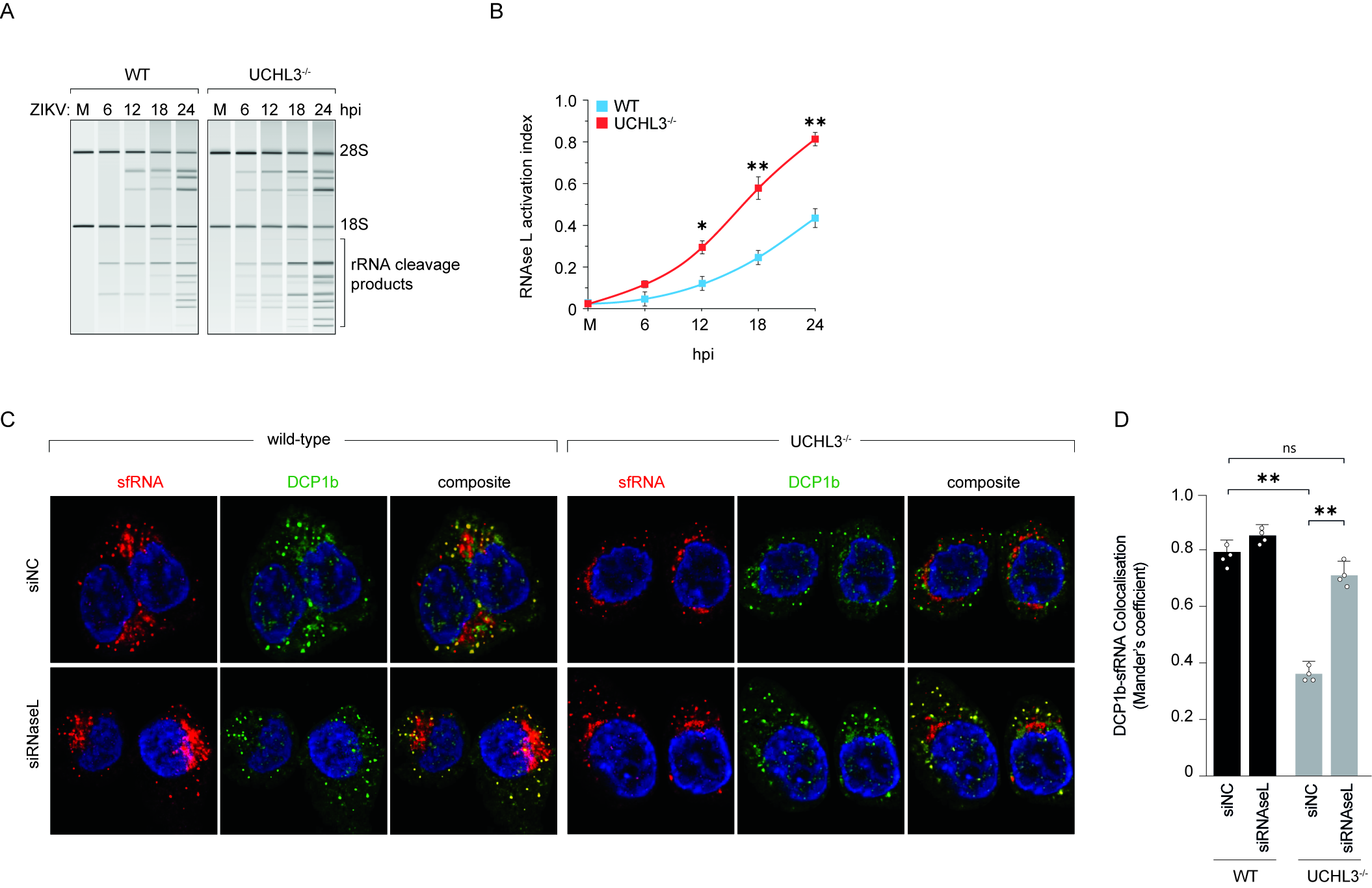

Supplement: Supplementary file 7 — Supporting File: advs75949‐sup‐0007‐FigureS4.tif. [file ADVS-9999-e21781-s006.tif]
